# SYVN1-mediated ubiquitylation directs localization of MCT4 in the plasma membrane to promote the progression of lung adenocarcinoma

**DOI:** 10.1038/s41419-023-06208-x

**Published:** 2023-10-10

**Authors:** Meng Zhao, Chen Huang, Lexin Yang, Boyu Pan, Shuting Yang, Jiao Chang, Yu Jin, Gang Zhao, Dongsheng Yue, Shuo Qie, Li Ren

**Affiliations:** 1 Department of Clinical Laboratory, Tianjin Medical University Cancer Institute & Hospital, Tianjin, China; 2 National Clinical Research Center for Cancer, Tianjin, China; 3 Key Laboratory of Cancer Prevention and Therapy (Tianjin), Tianjin, China; 4 Tianjin’s Clinical Research Center for Cancer, Tianjin, China; 5 Department of Molecular Pharmacology, Tianjin Medical University Cancer Institute & Hospital, Tianjin, China; 6 Department of Pathology, Tianjin Medical University Cancer Institute & Hospital, Tianjin, China; 7 Department of Lung Cancer, Tianjin Medical University Cancer Institute & Hospital, Tianjin, China

**Keywords:** Cancer metabolism, Non-small-cell lung cancer

## Abstract

Tumour cells mainly generate energy from glycolysis, which is commonly coupled with lactate production even under normoxic conditions. As a critical lactate transporter, monocarboxylate transporter 4 (MCT4) is highly expressed in glycolytic tissues, such as muscles and tumours. Overexpression of MCT4 is associated with poor prognosis for patients with various tumours. However, how MCT4 function is post-translationally regulated remains largely unknown. Taking advantage of human lung adenocarcinoma (LUAD) cells, this study revealed that MCT4 can be polyubiquitylated in a nonproteolytic manner by SYVN1 E3 ubiquitin ligase. The polyubiquitylation facilitates the localization of MCT4 into the plasma membrane, which improves lactate export by MCT4; in accordance, metabolism characterized by reduced glycolysis and lactate production is effectively reprogrammed by SYVN1 knockdown, which can be reversed by MCT4 overexpression. Biologically, SYVN1 knockdown successfully compromises cell proliferation and tumour xenograft growth in mouse models that can be partially rescued by overexpression of MCT4. Clinicopathologically, overexpression of SYVN1 is associated with poor prognosis in patients with LUAD, highlighting the importance of the SYVN1-MCT4 axis, which performs metabolic reprogramming during the progression of LUAD.

## Introduction

Metabolic reprogramming plays critical roles in tumorigenesis and tumour progression [1]. Tumour metabolism exhibits two major characteristics, the Warburg effect and active glutaminolysis [2]. The Warburg effect defines tumour cells prefer gaining energy through glycolysis, which is commonly coupled to lactic acid production even in the presence of oxygen. Lactate is a waste product of glycolysis; however, lactate and acidosis as a result of increased lactate levels in carcinogenesis are gaining increased attention. For example, lactate is gaining attention as an important tumour-promoting metabolite. Increased lactate levels in the tumour microenvironment facilitate multiple oncogenic, lactate-stimulated processes, such as the hypoxic response, neoangiogenesis, invasion and metastasis [3–6]. In addition, lactate-induced acidification of the microenvironment leads to evasion of the immune response in tumours [7]. Moreover, lactate can act as an extracellular ligand by binding to its receptor, GPR81, at the cell surface. GPR81, as a G_i_-coupled receptor for lactate, is commonly expressed in various tumour cells. The activation of GPR81 signalling tunes the lactate-sensitive machinery to maintain tumour growth and metastasis by activating the PD-L1/PD-1 immune checkpoint, which finally compromises immune surveillance [8, 9]. Given the involvement of lactate in supporting tumour initiation and progression, targeting aberrant lactate metabolism in tumour cells is a promising approach to treat cancers. Therefore, delineating the detailed mechanisms of how lactate metabolism is regulated is a prerequisite to develop strategies through targeting lactate metabolism and relevant signalling.

Tumour cells export lactate to survive the accumulation of lactate and to maintain intracellular acid‒base homeostasis. In most mammalian cells, the transport of lactate is mediated by members of the monocarboxylate transporter (MCT) family. These passive transporters are localized at the plasma membrane and move monocarboxylate ions together with protons bidirectionally depending on the concentration gradient of their substrates [10]. Among the four MCTs, MCT4 is mainly expressed in glycolytic tissues, such as muscles and tumour tissues [10–13], and overexpression of MCT4 is associated with poor prognosis in patients with a plethora of tumours [14–16], including non-small cell lung cancer (NSCLC), breast cancer, cervical cancer, colorectal cancer, oesophageal adenocarcinoma, hepatocellular carcinoma and melanoma [17]. Mechanistically, MCT4 is upregulated by hypoxia-inducible factor-1 (HIF-1) to enhance the cellular efflux of lactic acid/H^+^ [10, 18]. Therefore, inhibiting the expression or blocking the functions of MCT4 may be promising for a wide variety of neoplasms [11, 19, 20]. However, whether MCT4 function is regulated at the posttranslational level remains unknown. Thus, clarifying posttranslational modifications may provide new insights into targeting MCT4.

Protein ubiquitylation regulates a plethora of biological functions that depend on the specific target residues in the modified proteins and the nature of the ubiquitin chain linkages [21, 22]. Ubiquitylation can target proteins to the proteasome or lysosome for degradation or regulate protein interactions, activity and localization through nonproteolytic mechanisms [23]. Ubiquitylation is catalysed by a cascade of enzymes, including ubiquitin-activating enzyme (E1), ubiquitin-conjugating enzyme (E2), and ubiquitin ligase (E3) [24]. Synoviolin 1 (SYVN1), also known as 3-hydroxy-3-methylglutaryl reductase degradation (HRD1), is a RING domain E3 ubiquitin ligase. As a component of the ER-associated degradation (ERAD) system, SYVN1 resides in the ER membrane and catalyses the polyubiquitylation and degradation of a subset of ERAD-targeted proteins when unfolded/misfolded, such as p53, AMFR/Gp78, NRF2, and NRF3. [25]. Whether SYVN1 can mediate nonproteolytic ubiquitylation of its substrates in addition to protein degradation remains unknown. Notably, several studies have revealed that SYVN1 can mediate the ubiquitylation of multiple metabolic enzymes, such as PFKP, CPTII, GLUT1, and CD147, to maintain energy and metabolic homeostasis [26–28]; therefore, SYVN1 is a critical regulator of metabolic reprogramming.

In this study, MCT4 was found to undergo polyubiquitylation that promotes the localization of MCT4 in the plasma membrane, where MCT4 performs its function to export lactate. Importantly, SYVN1 was identified as an E3 ubiquitin ligase that catalyses the nonproteolytic ubiquitylation of MCT4, which regulates glycolytic metabolism and thus controls cell proliferation and the progression of lung adenocarcinoma (LUAD).

## Materials and methods

Other Materials and method details are presented in the Supplementary Information file.

### Animal study

The animal studies were reviewed and approved by the Ethics Committee for Animal Studies at Tianjin Medical University Cancer Institute & Hospital (No.: NSFC-AE-2021195). Thirty female BALB/c nude mice were purchased from Beijing Vital River Laboratory Animal Technologies. All mouse studies were approved by the Animal Ethics Committee of Tianjin Medical University Cancer Institute and Hospital. All mice were 5–6 weeks of age at the time of injection. The mice were randomly divided into three groups (ten mice per group). For animal experiments, no blinding was performed. A549-scramble, A549-shSYVN1-1, and A549-shSYVN1-2 cells were trypsinized into single-cell suspensions and resuspended in PBS. Approximately 5 × 10^6^ cells in 100 µl were subcutaneously injected into the right flanks. Mice were euthanized 3–4 weeks after inoculation. Then, the weight of the subcutaneous tumours was recorded and used to determine tumour growth.

### Clinical specimen

In this study, 20 sets of NSCLC tissue samples and their normal counterparts were obtained from patients at Tianjin Medical University Cancer Institute & Hospital. The tissues were evaluated by two independent pathologists who were blinded to the experiment. Patients who presented other malignancies with incomplete clinicopathologic data were excluded from this study. The studies involving human specimens were reviewed and approved by the Tianjin Medical University Cancer Institute & Hospital (No.: bc2019038). All participants signed an informed consent form.

### Nuclear magnetic resonance analysis

NMR analysis was performed with human NSCLC tissues and mouse tumour tissues from the A549-scramble and A549-shSYVN1 groups by Protein T (Tianjin). All participants signed an informed consent form. The tissue samples were cut into small pieces on dry ice, and 50 ± 5 mg of each sample was weighed for further extraction. Extraction buffer (0.6 mL, V_methanol_ : V_water_ 2:1, precooled at −20 °C overnight) was mixed with the sample, and 5 min (2 s-on, 3 s-off) of ultrasonication was performed to release the metabolites. The samples were then centrifuged (4 °C, 12,000 *g*, 10 min) to collect the supernatant. The sediments were further ultrasonicated for 5 min (2 s-on, 3 s-off) with 0.6 mL of extraction buffer and centrifuged (4 °C, 12,000 *g*, 10 min) to collect the supernatant twice. The supernatant was combined and centrifuged at 4 °C and 16,099 *g* for 10 min to collect the supernatant. The supernatant samples were dried by lyophilization, and the dried pellets were weighed. The pellets were dissolved in 0.6 mL detection buffer (75 mM Na_2_HPO_4_, 2 mM NaN_3_, 4.6 mM sodium trimethylsilyl propionate-[2,2,3,3-2H_4_] (TSP) in 80% D_2_O, pH 7.4 ± 0.1) and centrifuged at 4 °C and 16,099 *g* for 10 min. Then, 550 µL of the mixture was transferred into a Bruker SampleJetTM NMR tube (5 mm) and sealed with POM balls added to the caps.

NMR measurements were performed on Bruker 600 MHz Avance III HD spectrometers (IVDr) equipped with BBI probes and fitted with Bruker SampleJetTM robots with the cooling system set to 5 °C. A quantitative calibration was completed prior to the analysis. All samples were analysed with the 12.5-min method using the Bruker in vitro Diagnostics research (IVDr) methods, and the report data were generated using Bruker IVDr Lipoprotein Subclass Analysis (B.I. LISATM) method. The standards of the target metabolites were dissolved in the detection buffer separately and detected with the same method as the samples to normalize the reported quantification value.

### In situ proximity ligation assay

The Duolink In Situ Red Starter Kit (DUO92101, Sigma‒Aldrich) was used according to the manufacturer’s instructions. The cells were cultured on coverslips in 24-well plates. For ubiquitin detection, the cells were transfected with Flag-MCT4-WT, Flag-MCT4-ΔC or Flag-MCT4-C only, fixed with 4% paraformaldehyde for 15 min, and permeabilized with 0.2% Triton X-100. After being blocked with Duolink blocking solution for 1 h at 37 °C, the cells were incubated with primary antibodies against Flag (dilution 1:200) and ubiquitin (dilution 1:50) at 4 °C overnight. After three washes in Buffer A, the PLA probe solution was applied and incubated for 1 h at 37 °C. Subsequently, the cells were incubated in ligation buffer for 30 min at 37 °C, and the amplification solution was added for 100 min at 37 °C. Then, the slides were mounted using Duolink in situ mounting medium containing DAPI for 15 min. Images were obtained by laser scanning confocal microscopy (Zeiss LSM-880) and analysed using ImageJ. For interaction with MCT4 and SYVN1, A549/H1752 cells were fixed, washed, permeabilized and blocked similarly and then incubated with anti-MCT4 (dilution 1:50) and anti-SYVN1 (dilution 1:50) at 4 °C overnight. Subsequent steps were performed as described above.

### Immunoprecipitation

For nondenaturing IP, cells were lysed with native lysis buffer (Solarbio) containing 1 mM phenylmethylsulfonyl fluoride (PMSF) and protease inhibitor cocktail (Sigma‒Aldrich) for 1 h at 4 °C. Lysates were clarified by centrifugation at 12,000 rpm for 10 min at 4 °C and then incubated with anti-Flag M2 beads (Sigma-Aldrich) overnight at 4 °C. For denaturing conditions, cells were lysed in EBC lysis buffer (50 mM Tris-HCL, pH 7.5, 120 mM NaCl, 1 mM EDTA, 0.5% NP40) containing 4% SDS and protease inhibitor cocktail and heated at 95 °C for 12 min to disrupt noncovalent interactions. After sonication, lysates were centrifuged at 13,000 rpm for 10 min at room temperature to remove precipitates, and the resultant supernatant was diluted to 0.4% SDS with the abovementioned lysis buffer. Lysates were incubated with anti-Flag M2 beads (Sigma) overnight at 4 °C, followed by five washes with lysis buffer. The proteins were eluted with 3 × Flag peptide and analysed by immunoblotting.

### In vivo ubiquitylation assay

For the in vivo ubiquitylation assay using Ni-NTA beads, the cells were transfected with His-ubiquitin and Flag-MCT4. Then, the transfected cells were lysed in denaturing buffer A (6 M guanidine-HCl, 0.1 M Na_2_HPO_4_/NaH_2_PO_4_, 10 mM imidazole, pH 8.0), and the ubiquitylated proteins were pulled down by Ni-NTA beads. Beads were then collected and sequentially washed with buffer A, buffer B (8 M urea, 0.1 M Na_2_HPO_4_/NaH_2_PO_4_, pH 8.0, 0.01 M Tris-HCl, pH 8.0, 25 mM imidazole) five times. The proteins were incubated with 60 μl elution buffer (8 M urea, 0.1 M Na_2_HPO_4_/NaH_2_PO_4_, 0.15 M Tris-HCl, pH 6.7, 200 mM imidazole). After elution, ubiquitylation of MCT4 was detected by immunoblotting using an anti-Flag antibody. For using anti-Flag M2 beads, the cells transfected with Flag-MCT4 were lysed in EBC buffer containing 4% SDS. Then, IP and western blotting were performed as previously described. Ubiquitination of MCT4 was detected by immunoblotting using an anti-ubiquitin antibody.

### Statistical analysis

Quantification and statistical analysis GraphPad Prism 8 software were used for data analysis. All experiments were performed with biological replicates and repeated at least three times. All data were normally distributed, and variance was similar between the groups that were statistically compared. Data are shown as the mean ± SD. Two-tailed Student’s t test was used for two-group comparisons. ANOVA with Bonferroni’s correction was used to compare multiple groups. A *P* value of less than 0.05 was considered statistically significant. In the graphed data, *P values < 0.05 and **P values < 0.01. ns, not significant.

## Results

### MCT4 is ubiquitylated in LUAD cells

**Fig. 1:**
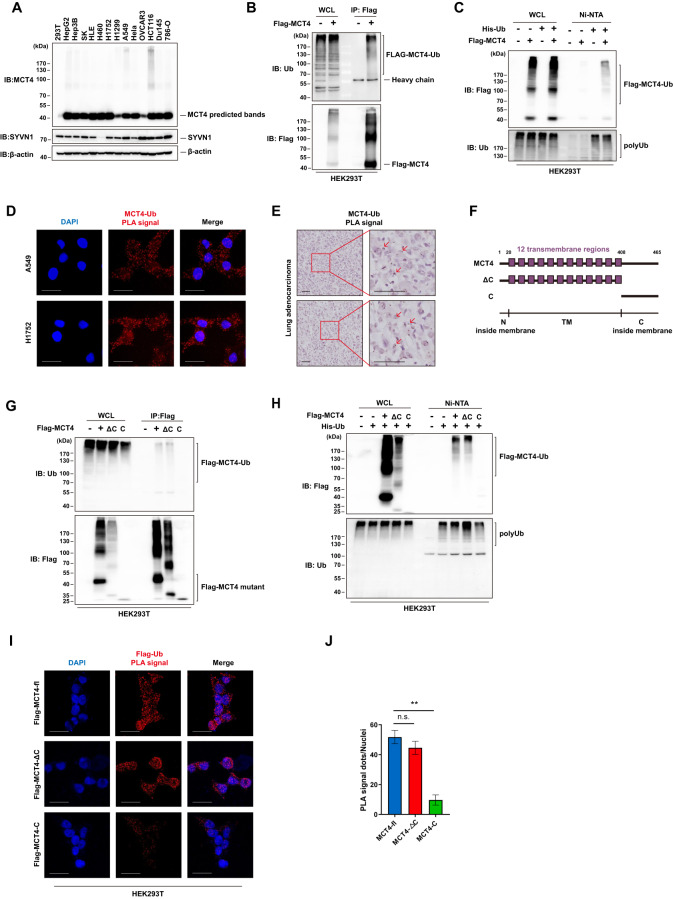
MCT4 is post-translationally modified by ubiquitylation in LUAD cells. **A** MCT4 expression pattern was detected by western blotting. Smeared bands were observed. **B** MCT4 ubiquitylation was detected in an in vivo ubiquitylation assay. Flag-tagged MCT4 was transfected into HEK293T cells. Cell lysates were immunoprecipitated with anti-Flag antibody under denaturing conditions. The immunoprecipitates were resolved and analysed by western blotting. **C** The ubiquitylation of MCT4 was demonstrated by a reverse ubiquitylation assay. Flag-tagged MCT4 was transfected into HEK293T cells along with His-tagged ubiquitin. The ubiquitylated proteins were purified under denaturing conditions via Ni-NTA agarose beads and were resolved and analysed. **D** The ubiquitylation of MCT4 was visualized by an in-situ proximity ligation (PLA) assay with anti-MCT4 and anti-Ub antibodies. The PLA signal (red) represents the intensity of ubiquitylated MCT4. Blue, nucleus. Scale bar: 20 μm. **E** The PLA assay identifies the ubiquitylation of MCT4 in LUAD tissues. Scale bar: 50 μm. **F** Schematic diagram of the MCT4 molecule and its deletion mutants. **G** In vivo ubiquitylation assay using MCT4 deletion mutants. Flag-tagged MCT4 or its mutants were transfected into HEK293T cells. Cell lysates were subjected to denaturing immunoprecipitation and immunoblotted with the indicated antibodies. **H** Reverse ubiquitylation assay using MCT4 deletion mutants. Flag-tagged MCT4 or its mutants were transfected into HEK293T cells along with His-tagged ubiquitin. The ubiquitylated proteins were purified under denaturing conditions via Ni-NTA agarose beads and were resolved and analysed. **I** The ubiquitylation of Flag-MCT4 and its mutants in HEK293T cells was detected by PLA. Scale bar: 20 μm. **J** The quantification of the PLA signals measured in (I) was performed using ImageJ software. Data are presented as the mean ± SD (**P < 0.01, *n* = 5, two-tailed unpaired *t* tests).

Smear patterned bands were detected when blotting with MCT4 antibody in a panel of tested tumour cell lines (Fig. 1A). The smear patterned bands suggest the presence of posttranslational modifications, such as protein ubiquitylation. To identify the posttranslational modification that contributes to this smear, an in vivo ubiquitylation assay was performed by overexpressing MCT4 in HEK293T cells. This assay confirmed that protein ubiquitylation occurs when MCT4 is pulled down (Fig. 1B); in accordance, MCT4 is found in the pool of ubiquitylated proteins (Fig. 1C). To further verify the ubiquitylation of endogenous MCT4, an in-situ proximity ligation assay (PLA) was performed using MCT4 and ubiquitin antibodies in human LUAD cells. The ubiquitylation of MCT4 was visualized by the strong PLA signals shown in red “spots” (Fig. 1D). In addition, MCT4 ubiquitylation was also observed in lung tissues in patients with LUAD (Fig. 1E).

Structurally, the MCT4 protein comprises intracellular N-termini, 12 transmembrane (TM) helices, intracellular C-termini, and a cytosolic loop between the sixth and seventh TM domains (Fig. 1F). To examine MCT4 ubiquitylation in molecular detail, Flag-tagged C-termini (C only) and its deletion mutant (ΔC) were generated for further analysis (Fig. 1F). An in vivo ubiquitylation assay revealed comparable levels of ubiquitylation for purified full-length MCT4 (FL) and the ΔC mutant but not for the C-only mutant (Fig. 1G). Consistently, similar levels of MCT4-FL and the ΔC mutant were also found in the pool of ubiquitylated proteins but not for the C-only mutant (Fig. 1H). Moreover, the PLA assay showed that the ubiquitylation signals were significantly weaker for the C only mutant than the MCT4-FL and ΔC mutants (Fig. 1I, J). Taken together, these results indicate that MCT4 can be ubiquitylated mainly in the transmembrane domain rather than in the C-terminal intracellular domain.

### SYVN1 is a specific E3 ubiquitin ligase for MCT4

**Fig. 2:**
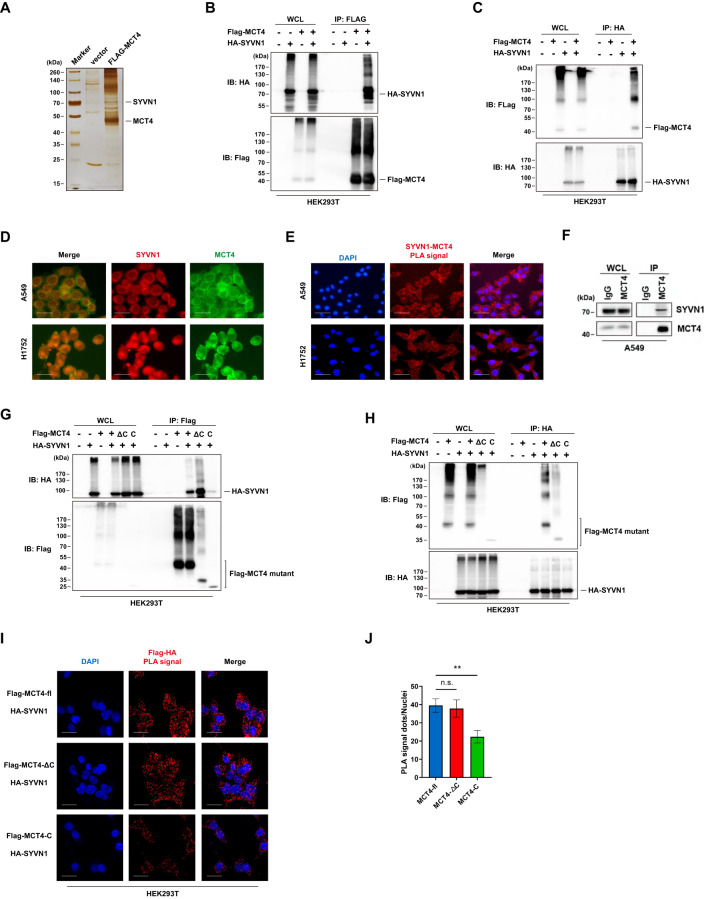
SYVN1 is physically associated with MCT4. **A** Immunopurification and mass spectrometry analysis of proteins associated with MCT4. Cellular extracts from Flag-MCT4-expressing HEK293T cells were purified with anti-Flag affinity beads and eluted with Flag peptide. The elutes were resolved by SDS‒PAGE and silver-stained. The protein bands were retrieved and analysed by mass spectrometry. **B, C** HEK293T cells were cotransfected with Flag-tagged MCT4 and HA-tagged SYVN1. Forty-eight hours post transfection, cell lysates were immunoprecipitated with anti-Flag antibody (**B**) or anti-HA antibody (**C**) and then immunoblotted with the indicated antibodies. **D** The colocalization of MCT4 and SYVN1 was determined by an immunofluorescence assay. Blue, nucleus; green, MCT4; red, SYVN1. Scale bar: 20 μm. **E** The interaction between MCT4 and SYVN1 was detected by a PLA assay. The PLA signal represents the intensity of the interaction between MCT4 and SYVN1. Blue, nucleus; red, PLA signal. Scale bar: 50 μm. **F** Immunoprecipitation analysis confirmed the interaction between endogenous SYVN1 and MCT4 in A549 cells. **G, H** HA-tagged MCT4 or its deletion mutants were transfected into HEK293T cells along with HA-tagged SYVN1. The cell lysates were immunoprecipitated with anti-Flag antibody (**G**) or anti-HA antibody (**H**) and then immunoblotted with the indicated antibodies. **I** The interaction between HA-tagged SYVN1 and Flag-tagged MCT4 or its deletion mutants was detected by the PLA assay in HEK293T cells cotransfected with HA-tagged SYVN1 and Flag-tagged MCT4 or its deletion mutants. The PLA signal represents the intensity of the interaction. Scale bar: 20 μm. **J** The PLA signals measured in (**I**) were analysed using ImageJ software. Data are presented as the mean ± SD. (**P < 0.01, *n* = 8, two-tailed unpaired *t* tests).

Immunoprecipitation combined with mass spectrometry analysis was employed to identify the specific E3 ubiquitin ligase of MCT4. LC‒MS/MS analysis revealed that SYVN1 was present in MCT4 immunoprecipitates (Fig. 2A). SYVN1, also termed HRD1, functions as a RING domain E3 ubiquitin ligase that catalyses the polyubiquitylation of ERAD-targeted proteins. Therefore, SYVN1 was selected for further analysis because it might function as a potential E3 ubiquitin ligase for MCT4. To confirm the MS results, HEK293T cells were cotransfected with HA-SYVN1 and Flag-MCT4 and then nondenaturing coimmunoprecipitation was performed. The results showed that MCT4 efficiently coimmunoprecipitated with SYVN1 (Fig. 2B); reciprocally, SYVN1 also efficiently pulled down MCT4 (Fig. 2C). To further validate their physical association, the colocalization of MCT4 and SYVN1 was examined using immunofluorescence staining in LUAD cells. The staining revealed that MCT4, as a monocarboxylate transporter, is predominantly localized in the plasma membrane, while SYVN1, as a component of the endoplasmic reticulum quality control (ERQC) system, is mainly observed in the cytoplasm; interestingly, some signal overlays are observed between MCT4 and SYVN1 in the cytoplasm (Fig. 2D). Accordingly, the PLA assay also revealed the colocalization of MCT4 and SYVN1 in the cytoplasm (Fig. 2E). Importantly, immunoprecipitation also revealed the interaction between endogenous SYVN1 and MCT4 (Fig. 2F).

Next, the association of MCT4 with SYVN1 was analysed to further consolidate their interaction and to gain molecular insights into the interaction between these two proteins. Coimmunoprecipitation analysis revealed that the transmembrane domain is indispensable for the interaction between MCT4 and SYVN1 (Fig. 2G, H). Consistently, the intensity and positive rate of the PLA signals originating from SYVN1 with the C-only mutant were significantly lower than those with MCT4-FL and the ΔC mutant (Fig. 2I, J). Collectively, these data suggest the molecular interface between MCT4 and SYVN1 in which MCT4 mainly binds to SYVN1 through its transmembrane domain, which is consistent with the ubiquitylation site assay.

### SYVN1 catalyses the nonproteolytic ubiquitylation of MCT4

**Fig. 3:**
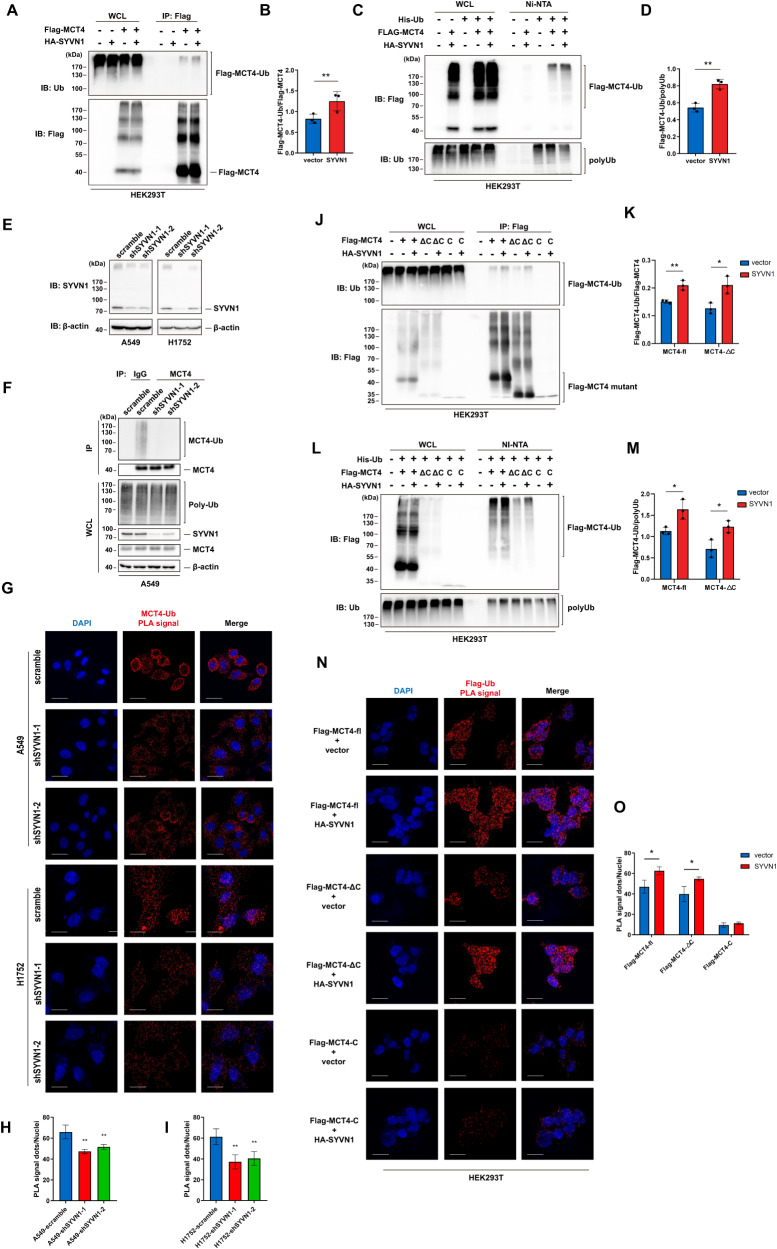
SYVN1 catalyses the ubiquitylation of MCT4. **A**, **B** In vivo ubiquitylation assay of MCT4 catalysed by SYVN1. Flag-tagged MCT4 was transfected into HEK293T cells along with HA-tagged SYVN1. Forty-eight hours post-transfection, cell lysates were immunoprecipitated with an anti-Flag antibody under denaturing conditions. The immunoprecipitates were analysed by immunoblotting with the indicated antibodies (**A**). **B** Amounts of ubiquitylated MCT4 in (**A**) were determined by densitometry of protein bands. Flag-MCT4 was used as the loading control. Data are presented as the mean ± SD. (**P < 0.01, *n* = 3, two-tailed unpaired *t* tests). **C**, **D** Reverse ubiquitylation assay of MCT4 catalysed by SYVN1. HEK293T cells were transfected with the indicated plasmids. Forty-eight hours post-transfection, the ubiquitylated proteins were purified under denaturing conditions via Ni-NTA agarose beads and analysed by immunoblotting with the indicated antibodies (**C**). **D** Amounts of ubiquitylated MCT4 in (**C**) were determined by densitometry of protein bands. PolyUb was used as the loading control. Data are presented as the mean ± SD. (**P < 0.01, *n* = 3, two-tailed unpaired *t* tests). **E** western blots show the knockdown of SYVN1. A549 and H1752 cells were infected with lentivirus carrying scramble control shRNA (scramble) or shRNAs targeting SYVN1. **F** In vivo ubiquitylation assay of endogenous MCT4 upon SYVN1 knockdown. A549 cells were transduced with scramble or SYVN1 shRNA lentiviral vector. Cell lysates were immunoprecipitated with anti-MCT4 antibody under denaturing conditions. The immunoprecipitates were resolved and analysed by western blotting. **G** The PLA assay demonstrates MCT4 ubiquitylation in cells with SYVN1 knockdown. Scale bar: 20 μm. **H**, **I** The quantification of the PLA signals measured in (**G**). Data are presented as the mean ± SD (**P < 0.05, *n* = 6, two-tailed unpaired *t* tests). **J** HEK293T cells were transfected with the indicated plasmid. Forty-eight hours post transfection, cell lysates were immunoprecipitated with an anti-Flag antibody under denaturing conditions. The immunoprecipitates were analysed by immunoblotting with the indicated antibodies. **K** Amounts of ubiquitylated MCT4 and its mutants in (**I**) were quantified by densitometry of protein bands. Flag-MCT4 was used as the loading control. Data are presented as the mean ± SD. (**P < 0.01, *n* = 3, two-tailed unpaired *t* tests). **L** HEK293T cells were transfected with the indicated plasmids. Forty-eight hours post transfection, the ubiquitylated proteins were purified under denaturing conditions via Ni-NTA agarose beads and analysed by immunoblotting with the indicated antibodies. **M** Amounts of ubiquitylated MCT4 and its mutants in (**L**) were quantified by densitometry of protein bands. PolyUb was used as the loading control. Data are presented as the mean ± SD. (**P < 0.01, n = 3, two-tailed unpaired *t* tests). **N** The PLA assay demonstrates the ubiquitylation of MCT4 and its deletion mutants upon SYVN1 overexpression. Scale bar: 20 μm. **O** Quantification of the PLA signals measured in (**N**). Data are presented as the mean ± SD (**P < 0.01, *n* = 6, two-tailed unpaired *t* tests).

To test whether SYVN1 can catalyse the ubiquitylation of MCT4, an in vivo ubiquitylation assay was performed in HEK293T cells overexpressing SYVN1. The results showed that SYVN1 enhanced MCT4 ubiquitylation when total MCT4 was used as a control for normalization (Fig. 3A, B). Accordingly, increased MCT4 was also found in the pool of ubiquitylated proteins (Fig. 3C, D). To consolidate these observations, lentiviral shRNAs were utilized to knockdown SYVN1 in LUAD cells (Fig. 3E). Not surprisingly, SYVN1 knockdown effectively reduced MCT4 ubiquitylation (Fig. 3F). Consistently, upon SYVN1 knockdown, a reduced MCT4-ubiquitin signal was demonstrated when analysed by the PLA assay, highlighting that SYVN1 is necessary for MCT4 ubiquitylation (Fig. 3G–I). To further investigate the region that determines the ubiquitylation of MCT4 by SYVN1, an in vivo ubiquitylation assay was performed using Flag-tagged MCT4-FL or deletion mutants (ΔC and C only). Overexpression of SYVN1 led to increased ubiquitylation of MCT4-FL and the ΔC mutant but not of the C-only mutant (Fig. 3J–M). Accordingly, the PLA assay also demonstrated that PLA signals were enhanced for MCT4-FL and the ΔC mutant but not for the C-only mutant (Fig. 3N, O). Taken together, these results indicate that SYVN1 catalyses MCT4 ubiquitylation in the transmembrane domain of MCT4.

### Ubiquitination of MCT4 determines its localization in the plasma membrane

**Fig. 4:**
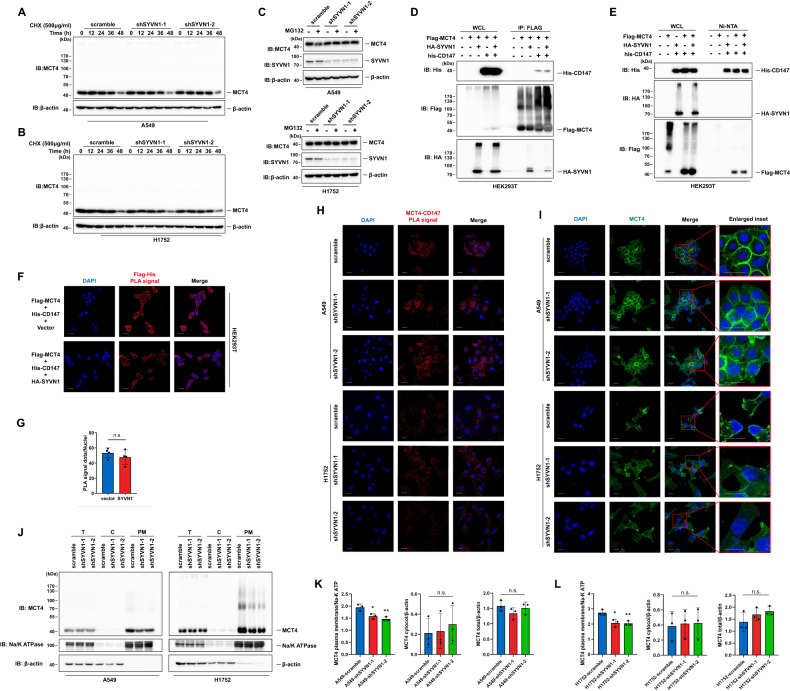
Ubiquitination of MCT4 determines its localization in the plasma membrane. **A**, **B** SYVN1 does not affect the protein stability of MCT4. A549 and H1752 cells infected with lentiviruses carrying the indicated shRNAs were treated with CHX (500 μg/ml) and harvested at the indicated time followed by western blotting analysis. **C** MG132 treatment does not alter MCT4 abundance. A549 and H1752 cells infected with lentiviruses carrying the indicated shRNAs were treated with MG132 (10 μM) for 8 h. **D**, **E** HEK293T cells were transfected with the indicated plasmids. Forty-eight hours post transfection, cell lysates were immunoprecipitated with anti-Flag antibody (**D**) or pulled down by Ni-NTA agarose beads (**E**) and then immunoblotted with the indicated antibodies. **F** HEK293T cells were transfected with the indicated plasmids. The interaction between MCT4 and CD147 was detected by a PLA assay with anti-Flag and anti-His antibodies. The PLA signal represents the intensity of the interaction. Scale bar: 20 μm. **G** Quantification of the PLA signals measured in (**F**) was performed using ImageJ software. Data are presented as the mean ± SD. (n.s., no significance, *n* = 4, two-tailed unpaired *t* tests). **H** The interaction between MCT4 and CD147 did not obviously change based on the PLA assay. Blue, nucleus. Scale bar: 20 μm. **I** The subcellular localization of MCT4 (green) in cells infected with lentivirus carrying scramble control shRNA (scramble) or shRNAs targeting SYVN1 was determined by an immunofluorescence assay. Blue, nucleus. Scale bar: 20 μm. **J** A549 and H1752 cells were infected with lentivirus carrying scramble control shRNA (scramble) or shRNAs targeting SYVN1. Total cell lysate (T), cytosol fraction C and plasma membrane fraction (PM) were analysed by immunoblotting with the indicated antibodies. **K**, **L** Quantification of MCT4 signals in different cellular fractions. (*P < 0.05, **P < 0.01, *n* = 3, two-tailed unpaired *t* test).

To further dissect the functional importance of MCT4 ubiquitylation, the effects of SYVN1 on MCT4 protein levels were first examined. A cycloheximide (CHX) chase assay showed that knockdown of SYVN1 did not alter the half-life of the MCT4 protein (Fig. 4A, B), suggesting that SYVN1 exhibited no effect on the protein stability of MCT4. Instead, treatment with MG132, a proteasome inhibitor, did not alter MCT4 abundance (Fig. 4C), suggesting that SYVN1 catalyses the nonproteolytic ubiquitylation of MCT4. Consistently, no negative correlation was detected for SYVN1 and MCT4 protein levels (Supplementary Fig. 1). Normally, nonproteolytic ubiquitylation controls the status of signalling pathways; for example, K63-linked ubiquitylation regulates the activity of YAP and Akt signalling. CD147 functions as a molecular chaperone of MCT4, and the interaction between these two proteins is critical for lactate export. To address whether the interaction between MCT4 and CD147 is affected by SYVN1-mediated ubiquitylation, HEK293T cells were cotransfected with Flag-MCT4 and HA-CD147, and a coimmunoprecipitation assay was performed to investigate the status of the physical interaction between these two proteins. Surprisingly, SYVN1 overexpression did not alter their interaction (Fig. 4D, E). Accordingly, the PLA assay showed that neither overexpression nor knockdown of SYVN1 changed the colocalization signal of these two proteins (Fig. 4F–H), further confirming that MCT4 ubiquitylation does not affect the formation of a heterodimeric complex with CD147. However, immunofluorescence revealed that knockdown of SYVN1 effectively attenuated the localization of MCT4 in the plasma membrane while enhancing its localization in the cytoplasm (Fig. 4I). Consistently, immunoblotting demonstrated that SYVN1 knockdown also compromises MCT4 expression in the plasma membrane (Fig. 4J–L), suggesting that an unknown mechanism occurs that facilitates the localization of MCT4 in the plasma membrane upon ubiquitylation.

### SYVN1 enhances glycolysis and lactate export

**Fig. 5:**
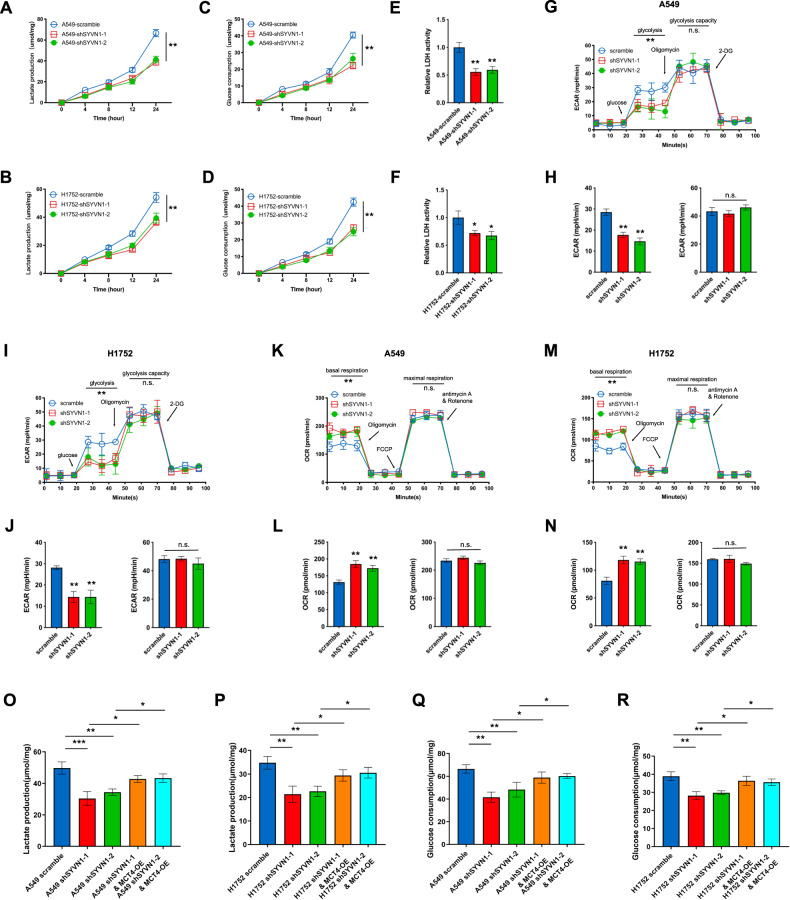
SYVN1 enhances glycolysis and lactate export in LUAD cells. **A**–**D** Lactate production and glucose consumption were monitored at the indicated time points in A549 and H1752 cells infected with lentiviruses carrying scramble shRNA (scramble) or shRNAs targeting SYVN1. Data are presented as the mean ± SD (**P < 0.01, *n* = 5, two-way ANOVA). **E**, **F** LDH activity is compromised in A549 (**E**) and H1752 (**F**) cells upon SYVN1 knockdown. Data are presented as the mean ± SD (*P < 0.05, **P < 0.01, *n* = 3, two-tailed unpaired *t* test). **G**–**J** SYVN1 knockdown alters the extracellular acidification rate (ECAR). A549 and H1752 cells were infected with lentiviruses carrying the indicated shRNA. The ECAR was assessed after glucose was added, oligomycin (oligo), and 2-deoxyglucose (2DG). **G**, **I** Time course of a representative experiment. **H**, **J** Determination of the glycolysis rate and glycolytic capacity. Data are presented as the mean ± SD (*P < 0.05, **P < 0.01, *n* = 3, two-tailed unpaired *t* test). **K**–**N** SYVN1 knockdown alters the oxygen consumption rate (OCR). A549 and H1752 cells were infected with lentiviruses carrying the indicated shRNA. OCR was measured after oligomycin, FCCP, and rotenone were added. **K**, **M** time course of a representative experiment. **L**, **N** determination of the OCR used for basal respiration and maximal respiration. Data are presented as the mean ± SD (*P < 0.05, **P < 0.01, *n* = 3, two-tailed unpaired *t* test). **O**, **R** Overexpression of MCT4 reverses lactate production (**O**, **P**) and glucose consumption (**Q**, **R**) in A549 and H1752 cells infected with scramble or shRNAs targeting SYVN1. OE indicates overexpression. Data are presented as the mean ± SD (*P < 0.05, **P < 0.01, *n* = 5, two-tailed unpaired *t* tests).

Increased glucose consumption and lactate production are the major characteristics of the Warburg effect. The cellular efflux of lactic acid/H^+^ is mainly controlled by MCT4 [12]. It remains elusive whether SYVN1-mediated ubiquitylation affects the metabolic phenotype of LUAD cells by regulating the activity of MCT4. The enzymatic assay showed that SYVN1 knockdown efficiently reduced lactate export compared with that in the control group (Fig. 5A, B). Importantly, SYVN1 knockdown cells exhibited a significant decrease in glucose consumption (Fig. 5C, D), suggesting the presence of reduced aerobic glycolysis. Lactate dehydrogenase (LDH) catalyses the conversion of pyruvate to lactate. In accordance with the reduced lactate export, SYVN1 knockdown cells also exhibited lower LDH activity than that of control cells (Fig. 5E, F). To obtain a detailed metabolic profile of SYVN1 knockdown cells, Seahorse analysis was performed to monitor the extracellular acidification rate (ECAR) and oxygen consumption rate (OCR), which are indicative of aerobic glycolysis and mitochondrial respiration, respectively. When glucose was added to enable glycolysis, a significant decrease in the ECAR was observed upon SYVN1 knockdown, suggesting that SYVN1 is a critical factor that controls basal glycolysis (Fig. 5G–J). However, upon oligomycin treatment, no difference in the ECAR was revealed in either SYVN1 knockdown or control cells, indicating that the cells exhibit equivalent maximum capacities for glycolysis (Fig. 5G–J). Compared with control cells, SYVN1 knockdown cells demonstrated decreased OCR, indicating impaired basal respiration (Fig. 5K–N). The same level of OCR was reached by SYVN1 knockdown and control cells upon treatment with FCCP, an uncoupling agent that collapses the proton gradient and disrupts the mitochondrial membrane potential (Fig. 5K–N); thus, maximal respiration was comparable in both cell lines. Importantly, overexpression of MCT4 successfully reversed lactate production (Fig. 5O, P) and glucose consumption (Fig. 5Q, R) upon SYVN1 knockdown. Moreover, we evaluated changes in the intracellular lactate and glucose levels. The results showed that overexpression of MCT4 can partially reverse lactate production (Supplementary Fig. 2A, B) and glucose consumption (Supplementary Fig. 2C, D) upon SYVN1 knockdown, which is consistent with their extracellular level changes; this result further highlights the importance of the SYVN1-MCT4 axis in regulating metabolic reprogramming.

### SYVN1 promotes LUAD progression through metabolic reprogramming

**Fig. 6:**
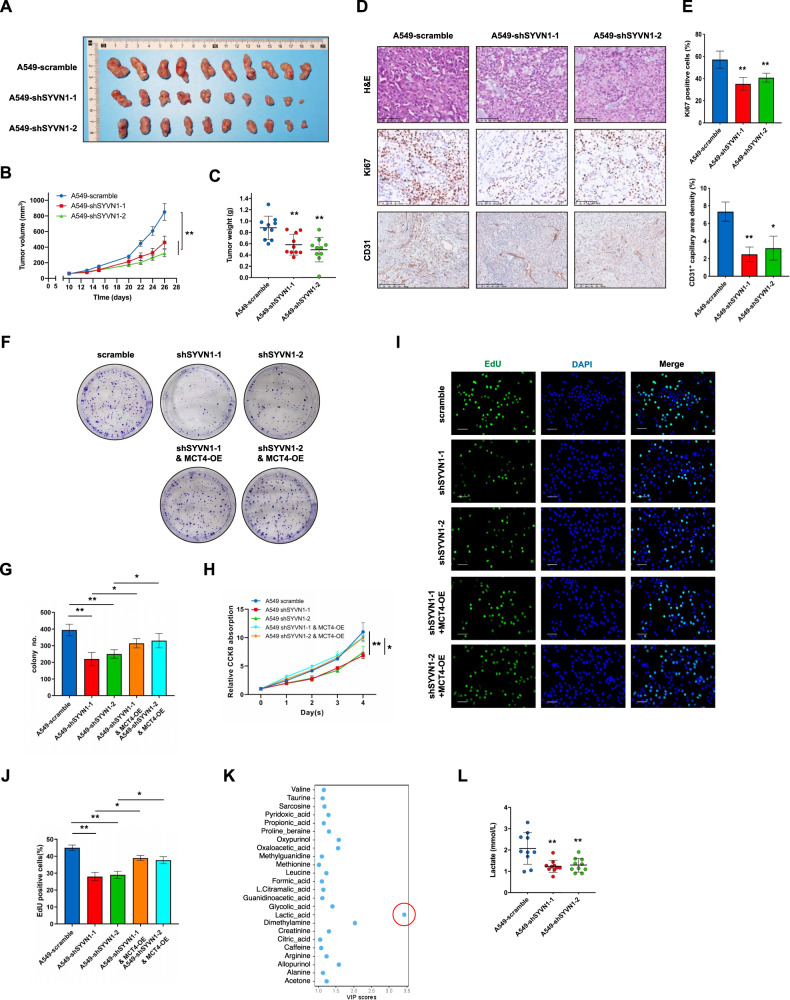
SYVN1 promotes LUAD progression through metabolic reprogramming. **A**–**C** A tumour formation assay was performed by injecting A549 cells infected with lentiviruses carrying scramble control shRNA (scramble) or shRNAs targeting SYVN1. Tumour volumes (**A**) and weights (**B**) were measured. Data are presented as the mean ± SD (**P < 0.01, *n* = 10, two-tailed unpaired t test for tumour weight analysis and two-way ANOVA for tumour volume analysis). **D**, **E** Representative images of IHC of the cell proliferation marker Ki67 and capillary marker CD31 in tumour sections from the xenograft models at the endpoint. The histogram shows the quantification of Ki67-positive cells and CD31+ capillary area density. Data are presented as the mean ± SD (*P < 0.05, **P < 0.01, *n* = 10 mice, two-tailed unpaired *t* test). Scale bar: Ki67, 100 μm; CD31, 250 μm. **F**, **G** Overexpression of MCT4 partially reverses the SYVN1 knockdown-induced inhibition of cell proliferation. Colony formation of SYVN1 scramble and SYVN1 knockdown with overexpression of MCT4 in A549 cells. (**G**) Data are quantified and presented as the mean ± SD (*P < 0.05, **P < 0.01, *n* = 5, two-tailed unpaired *t* test). **H** CCK8 assays of SYVN1 scramble and SYVN1 knockdown with overexpression of MCT4 in A549 cells. Data are presented as the mean ± SD (*P < 0.05, **P < 0.01, *n* = 5, two-way ANOVA). **I**, **J** EdU incorporation assays of SYVN1 scramble and SYVN1 knockdown with overexpression of MCT4 in A549 cells. **J** Data are quantified and presented as the mean ± SD (*P < 0.05, **P < 0.01, *n* = 6, two-tailed unpaired *t* test). Scale bar: 50 μm. **K** VIP values of different metabolites between A549 cells infected with lentiviruses carrying scramble control shRNA (scramble) or shRNAs targeting SYVN1 were detected by NMR analysis. **L** Lactate export in tumour tissues from A549 cells infected with lentiviruses carrying scramble control shRNA (scramble) or shRNAs targeting SYVN1 was detected by NMR analysis. Data are presented as the mean ± SD (**P < 0.01, *n* = 10 mice, two-tailed unpaired *t* test).

Since SYVN1 is strongly linked to tumour metabolic reprogramming and to increased lactate efflux from LUAD cells, investigating the role of SYVN1 in tumour progression is important. Colony formation assays showed that knockdown of SYVN1 significantly decreased colony formation in LUAD cells (Supplementary Fig. 3A, B). In addition, SYVN1 knockdown effectively compromised cell proliferation (Supplementary Fig. 3C) and the incorporation of EdU (Supplementary Fig. 3D, E), highlighting that SYVN1 is critical for DNA replication. To further verify the role of SYVN1 in LUAD progression, nude mouse xenografts were established. Notably, SYVN1 knockdown greatly reduced the tumour volume and tumour growth rate (Fig. 6A–C), as indicated by reduced Ki-67 staining (Fig. 6D, E). Interestingly, there was a dramatic reduction in microvessel density in xenografts with SYVN1 knockdown, as indicated by decreased CD31 (+) capillary structures (Fig. 6D, E). These results demonstrate that SYVN1 plays a tumour-progressive role. To further define whether MCT4 is involved in SYVN1-mediated LUAD progression, MCT4 was overexpressed in SYVN1-knockdown cells to perform rescue experiments. Overexpression of MCT4 partially reversed the inhibition of colony formation upon SYVN1 knockdown (Fig. 6F, G and Supplementary Fig. 4A, B). Accordingly, cell proliferation (Fig. 6H and Supplementary Fig. 4C) and EdU incorporation (Fig. 6I, J and Supplementary Fig. 4D, E) were also partially rescued, further establishing the functional connection between SYVN1 and MCT4.

Since SYVN1-mediated ubiquitylation regulates MCT4 activity, nuclear magnetic resonance (NMR) analysis was performed to examine the metabolic impacts of SYVN1 on xenograft tumour samples. Partial least-square-discriminant analysis (PLS-DA) was performed, which revealed an obvious separate metabolic trend in tumour samples compared to the SYVN1 knockdown and control samples (Supplementary Fig. 5A; Supplementary Table 1). The PLS-DA load diagram represents the contribution of metabolites to discriminate the sample groups. Metabolites provide a greater contribution towards distinguishing the sample groups when the distance from the centre point is higher. The results revealed that lactate provides an important contribution to discriminating all tested samples (Supplementary Fig. 5B; Supplementary Table 2). Consistent with the in vitro data, the variable importance in projection (VIP) value of lactate was the largest among the top metabolites (Fig. 6K; Supplementary Table 3). Compared with the control group, the SYVN1 knockdown group exhibited a significant reduction in lactate production (Fig. 6L). The above data suggest that SYVN1 can promote LUAD progression by enhancing lactate export.

### The SYVN1-MCT4 axis drives tumour progression in human tumours

**Fig. 7:**
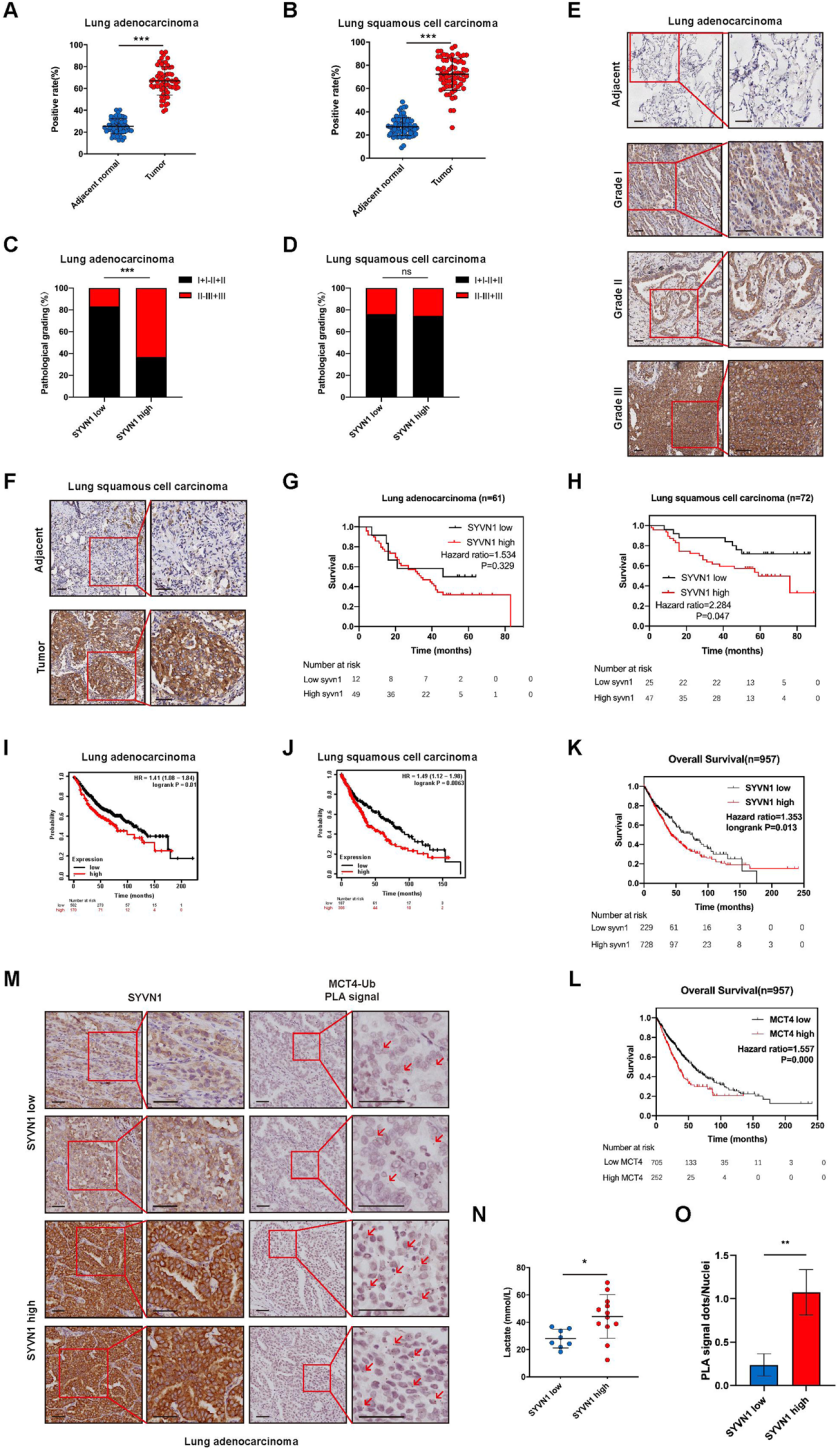
SYVN1 contributes to the progression of LUAD. **A**, **B** Comparison of SYVN1 expression in tumour and matched adjacent normal tissues in LUAD (**E**) and lung squamous cell carcinoma (**F**) by paired *t* test (***P < 0.001). **C**, **D** Correlation analysis between SYVN1 expression and pathological grading of patients with LUAD (**C**) and lung squamous cell carcinoma (**D**) by the Mann‒Whitney test (***P < 0.001). **E** The expression of SYVN1 in adjacent normal tissues and LUAD tissues with different pathological grades was determined by IHC staining; scale bar, 50 μm. **F** The expression of SYVN1 in adjacent normal tissues and lung squamous cell carcinoma tissues was determined by IHC staining; scale bar, 50 μm. **G**, **H** Overall survival rates of patients with low and high SYVN1 expression were determined in LUAD (**G**) and lung squamous cell carcinoma (**H**) by Kaplan‒Meier analysis. **I**, **J** KM plot analysis indicates the importance of SYVN1 expression in determining the prognosis of patients with LUAD (**I**) and lung squamous cell carcinoma (**J**) using KM plotter (https://kmplot.com/). **K**, **L** Overall survival rates of individuals with low and high SYVN1 (**K**) and MCT4 (**L**) expression levels were determined using TCGA datasets. **M** IHC staining of LUAD tissues with low and high SYVN1 expression. The ubiquitylation of MCT4 was detected by an in-situ proximity ligation assay (PLA) in LUAD tissues. Scale bar, 50 μm. **N** Lactate levels were determined by NMR analysis in tumour tissues with low and high SYVN1 expression. Data are presented as the mean ± SD (*P < 0.05, two-tailed unpaired *t* test). **O** The PLA signal represents the intensity of the ubiquitylation of MCT4 in LUAD tissues (**P < 0.01, *n* = 5, two-tailed unpaired *t* test).

To define the expression of SYVN1 in LUAD tissues, IHC analysis was performed in LUAD tissue microarrays, which included lung tumour tissues and their matched adjacent normal tissues. The positive rate of SYVN1 expression in LUAD and lung squamous cell carcinoma was significantly higher than that in adjacent normal tissues (Fig. 7A, B). Then, the correlation between the expression of SYVN1 and the pathological grade of LUAD was assessed using the Mann‒Whitney test. According to the intensity and density of positive cells, the IHC staining results indicated that the expression of SYVN1 was associated with pathological grading in LUAD but not in lung squamous cell carcinoma (Fig. 7C, D). Consistently, IHC staining demonstrated that LUAD tissues with high pathological grade exhibited much higher SYVN1 levels (Fig. 7E); moreover, SYVN1 was also overexpressed in lung squamous cell carcinoma compared with adjacent normal tissues (Fig. 7F). Kaplan‒Meier analysis indicated that high SYVN1 expression is correlated with poor overall survival in LUAD and lung squamous cell carcinoma based on our samples and data obtained from the KM plotter website (Fig. 7G–J). Next, the overall survival rates of LUAD and lung squamous cell carcinoma were evaluated according to SYVN1 and MCT4 expression independently using The Cancer Genome Atlas (TCGA) datasets (Fig. 7K, L). LUAD fresh specimens were collected for nuclear magnetic resonance (NMR) analysis. First, IHC analysis was performed to classify these tumours into two groups with low and high SYVN1 levels (Fig. 7M). Accordingly, elevated SYVN1 expression was correlated with lactate production (Fig. 7N), suggesting that SYVN1 can promote the progression of LUAD by enhancing lactate export.

To further investigate the correlation of SYVN1 and ubiquitylation of MCT4 in tumour progression, an in-situ proximity ligation assay for the ubiquitylation of MCT4 and IHC staining of SYVN1 were performed in LUAD. Compared with the tumours that exhibited high SYVN1 expression, the PLA signal of ubiquitylation of MCT4 was significantly decreased in tissues with low SYVN1 expression (Fig. 7M). These findings are consistent with the results obtained from cell culture, demonstrating that SYVN1 regulates MCT4 ubiquitylation. Furthermore, NMR analysis was also performed on tumours with different levels of MCT4 ubiquitylation (Fig. 7O). The results showed that tumours with higher MCT4 ubiquitylation produce more lactate. Taken together, these results suggest that SYVN1 may promote the progression of LUAD by increasing MCT4 ubiquitylation, finally leading to lactate export.

## Discussion

Lactate performs dual roles in metabolic pathways, as the compound is a final product of glycolysis and an energy-rich oxidative fuel. The production and efflux of lactate occur for the following major reasons: to prevent intracellular acidification and to regenerate NAD^+^. Intercellularly, lactate functions as a crosstalk nutrient that supports metabolic cooperation between cancer and host cells or between cancer cells with different metabolic characteristics [29]. The ability to precisely adjust lactate efflux is very important for tumour cells to survive, proliferate and migrate. The transport of lactate across the cellular membrane requires transporters, such as MCT4. To date, there is no detailed report on the posttranslational modification of MCT4. As such, this study identified that MCT4 can be ubiquitylated in established cell lines and samples from LUAD patients. This modification promotes the localization of MCT4 to the plasma membrane, therefore enhancing lactate export from LUAD cells. Mechanistically, as a membrane-integrated ubiquitin E3 ligase, SYVN1 was found to orchestrate MCT4 shuttling to the plasma membrane by catalysing the nonproteolytic ubiquitylation of MCT4. MCT4 ubiquitylation by SYVN1 promotes glycolysis and proliferation of LUAD cells in vitro and tumour growth in vivo, finally leading to tumour progression. This work reveals a previously uncharacterized modification of MCT4 in LUAD and undescribed functions of SYVN1 in cancer metabolism.

Although extensive attention has been placed on MCT4 in the field of tumour metabolism, knowledge of how MCT4 is regulated is mainly at the transcriptional level. For instance, hypoxia induces *SLC16A3* (MCT4) gene expression directly via the activation of HIF-1, which can bind to hypoxia response elements (HREs) in the *SLC16A3* promoter [12, 30]. Hypermethylation of the *SLC16A3* promoter leads to reduced MCT4 expression in colorectal carcinoma [31]. While this study focuses on the posttranslational modification of MCT4; in particular, MCT4 ubiquitylation is revealed in both established cell lines and NSCLC tumour samples. Notably, this modification is nonproteolytic, as it does not alter the expression levels or half-life of the MCT4 protein, but it efficiently affects the localization of MCT4 in the plasma membrane. Previous studies have demonstrated that CD147 can facilitate the localization of MCT4 into the plasma membrane [32]; however, our data revealed that ubiquitylation of MCT4 did not affect the interaction between CD147 and MCT4. Therefore, our findings provide new insight into the regulation of MCT4 subcellular localization and establish a firm foundation for further investigation. Importantly, it would be interesting to determine whether the subcellular localization of other MCTs is also regulated by protein ubiquitylation.

This study identified SYVN1 as a potential E3 ubiquitin ligase of MCT4, as evidenced by mass spectrometric analysis of the interacting partners of MCT4. Ubiquitination of MCT4 is compromised after SYVN1 knockdown and increased after SYVN1 overexpression, suggesting that SYVN1 is a bona fide E3 ubiquitin ligase for MCT4. SYVN1, which belongs to the RING domain E3 ubiquitin ligase family, is well known for its ability to mediate the degradation of unfolded/misfolded proteins in the ER, a process termed ERAD [33]. Previous studies have focused on the role of SYVN1 as a component of the E3 ubiquitin ligase complex in promoting proteolytic ubiquitylation of its substrates [34]. However, our data indicate that SYVN1 performs a nonproteolytic function, suggesting that the SYVN1-mediated ubiquitylation of MCT4 does not affect the expression and half-life of MCT4 but enhances the translocation of MCT4 from the cytoplasm to the plasma membrane. Accordingly, knockdown of SYVN1 suppresses glycolysis and lactate export, which is supported by histological data in which concurrence of SYVN1 expression occurred with MCT4 ubiquitylation and lactate concentrations in tumour samples from NSCLC patients. SYVN1 acts as a membrane hub that organizes a large set of surrounding proteins to form the main structural component of a channel through which ERAD substrates are transported across the ER membrane [25]. Whether SYVN1 utilizes the ERAD system to mediate nonproteolytic ubiquitylation of MCT4 warrants further investigation.

Tumour cells regulate lactate flow by modulating the expression and activity of key enzymes that control lactate production and consumption, such as LDH and the pyruvate dehydrogenase complex (PDH). Another method is altering the expression of membrane transporters for lactate, such as MCT1 and MCT4 [10, 35]. This study provides an emerging role of posttranslational modification of membrane transporters in regulating lactate efflux. Consistently, SYVN1 induces a Warburg effect and enhances glycolysis and lactate production by ubiquitylating MCT4 in NSCLC cells. Interestingly, LDH activity was also compromised upon SYVN1 knockdown. Unlike inhibitors targeting MCT1, other inhibitors, such as AZD3965, are currently undergoing clinical trials for several types of cancers (ClinicalTrials.gov NCT01791595) [36, 37], MCT4 inhibitors remain in the developing phase [10, 38]. Nevertheless, the findings in this study suggest that inhibiting MCT4 ubiquitylation can be a new strategy to selectively target MCT4 and to treat metabolism-addicted tumours.

Increasing evidence suggests that SYVN1 is involved in the progression of various malignancies, such as tumours from the liver, breast, and prostate as well as lymphoma [28, 39–41]. This study adds LUAD to the growing list of human cancers that involve SYVN1. Importantly, SYVN1 was found to play a role in mediating the Warburg effect in LUAD. A recent study showed that deletion of SYVN1 in the liver resulted in a significant reduction in serum glucose levels after refeeding. Compared to control mice, SYVN1^Alb^ mice exhibited dramatically lower serum triglycerides levels and significant changes in the expression of a large number of genes critical for lipid and fatty acid metabolism [28]. Another study revealed that SYVN1 ubiquitylates CPT2 to inhibit fatty acid oxidation and tumorigenesis in triple-negative breast cancer [42]. In addition to our study, these studies suggest that SYVN1 functions as a potential metabolic regulator.

In summary, we discovered a previously undescribed mechanism by which polyubiquitylation of MCT4 promotes its plasma localization, lactate export, and cell proliferation. Moreover, we identified that the E3 ubiquitin ligase SYVN1 regulates the nonproteolytic ubiquitylation of MCT4 without altering its protein levels but promotes metabolic reprogramming and the progression of LUAD.

### Supplementary information

Supplementary Materials

Supplementary Table 1

Supplementary Table 2

Supplementary Table 3

Original Data File

Checklist

## Data Availability

The datasets used and/or analysed during the current study are available from the corresponding author on reasonable request.

## References

[1] Martinez-Reyes I, Chandel NS (2021). Cancer metabolism: looking forward. Nat Rev Cancer.

[2] Qie S, Yoshida A, Parnham S, Oleinik N, Beeson GC, Beeson CC (2019). Targeting glutamine-addiction and overcoming CDK4/6 inhibitor resistance in human esophageal squamous cell carcinoma. Nat Commun.

[3] Watson MJ, Vignali PDA, Mullett SJ, Overacre-Delgoffe AE, Peralta RM, Grebinoski S (2021). Metabolic support of tumour-infiltrating regulatory T cells by lactic acid. Nature.

[4] Wang K, Huang W, Chen R, Lin P, Zhang T, Ni YF (2021). Di-methylation of CD147-K234 Promotes the Progression of NSCLC by Enhancing Lactate Export. Cell Metab.

[5] Ippolito L, Morandi A, Giannoni E, Chiarugi P (2019). Lactate: A Metabolic Driver in the Tumour Landscape. Trends Biochem Sci.

[6] Perez-Tomas R, Perez-Guillen I. Lactate in the tumor microenvironment: an essential molecule in cancer progression and treatment. Cancers (Basel). 2020;12. 10.3390/cancers12113244 PMC7693872 33153193

[7] Certo M, Tsai C-H, Pucino V, Ho P-C, Mauro C (2020). Lactate modulation of immune responses in inflammatory versus tumour microenvironments. Nat Rev Immunol.

[8] Feng J, Yang H, Zhang Y, Wei H, Zhu Z, Zhu B (2017). Tumor cell-derived lactate induces TAZ-dependent upregulation of PD-L1 through GPR81 in human lung cancer cells. Oncogene.

[9] Brown TP, Ganapathy V (2020). Lactate/GPR81 signaling and proton motive force in cancer: Role in angiogenesis, immune escape, nutrition, and Warburg phenomenon. Pharmacol Therapeutics.

[10] Payen VL, Mina E, Van Hee VF, Porporato PE, Sonveaux P (2020). Monocarboxylate transporters in cancer. Mol Metab.

[11] Takenaga K, Koshikawa N, Akimoto M, Tatsumi Y, Lin J, Itami M (2021). MCT4 is induced by metastasis-enhancing pathogenic mitochondrial NADH dehydrogenase gene mutations and can be a therapeutic target. Sci Rep.

[12] Ullah MS, Davies AJ, Halestrap AP (2006). The plasma membrane lactate transporter MCT4, but not MCT1, is up-regulated by hypoxia through a HIF-1alpha-dependent mechanism. J Biol Chem.

[13] Manning Fox JE, Meredith D, Halestrap AP (2000). Characterisation of human monocarboxylate transporter 4 substantiates its role in lactic acid efflux from skeletal muscle. J Physiol.

[14] Fisel P, Kruck S, Winter S, Bedke J, Hennenlotter J, Nies AT (2013). DNA methylation of the SLC16A3 promoter regulates expression of the human lactate transporter MCT4 in renal cancer with consequences for clinical outcome. Clin Cancer Res.

[15] Baek G, Tse YF, Hu Z, Cox D, Buboltz N, McCue P (2014). MCT4 defines a glycolytic subtype of pancreatic cancer with poor prognosis and unique metabolic dependencies. Cell Rep.

[16] Todenhöfer T, Seiler R, Stewart C, Moskalev I, Gao J, Ladhar S (2018). Selective Inhibition of the Lactate Transporter MCT4 Reduces Growth of Invasive Bladder Cancer. Mol Cancer Ther.

[17] Meijer TWH, Schuurbiers OCJ, Kaanders JHAM, Looijen-Salamon MG, de Geus-Oei L-F, Verhagen AFTM (2012). Differences in metabolism between adeno- and squamous cell non-small cell lung carcinomas: spatial distribution and prognostic value of GLUT1 and MCT4. Lung Cancer.

[18] Dimmer KS, Friedrich B, Lang F, Deitmer JW, Bröer S (2000). The low-affinity monocarboxylate transporter MCT4 is adapted to the export of lactate in highly glycolytic cells. Biochem J.

[19] Kelsey R (2016). Prostate cancer: MCT4 is a novel target for prostate cancer. Nat Rev Urol.

[20] Choi SYC, Xue H, Wu R, Fazli L, Lin D, Collins CC (2016). The MCT4 gene: a novel, potential target for therapy of advanced prostate cancer. Clin Cancer Res.

[21] Qie S, Majumder M, Mackiewicz K, Howley BV, Peterson YK, Howe PH (2017). Fbxo4-mediated degradation of Fxr1 suppresses tumorigenesis in head and neck squamous cell carcinoma. Nat Commun.

[22] Qie S. The E3 ubiquitin ligase Fbxo4 functions as a tumor suppressor: its biological importance and therapeutic perspectives. Cancers (Basel). 2022;14. 10.3390/cancers14092133 PMC9101129 35565262

[23] Komander D, Rape M (2012). The ubiquitin code. Annu Rev Biochem.

[24] Oh E, Akopian D, Rape M (2018). Principles of ubiquitin-dependent signaling. Annu Rev Cell Dev Biol.

[25] Karamali N, Ebrahimnezhad S, Khaleghi Moghadam R, Daneshfar N, Rezaiemanesh A (2022). HRD1 in human malignant neoplasms: molecular mechanisms and novel therapeutic strategy for cancer. Life Sci.

[26] Chen L, Wei J, Zhu H, Pan H, Fang D (2020). Energy supplementation rescues growth restriction and female infertility of mice with hepatic HRD1 ablation. Am J Transl Res.

[27] Kim D, Langmead B, Salzberg SL (2015). HISAT: a fast spliced aligner with low memory requirements. Nat Methods.

[28] Wei J, Yuan Y, Chen L, Xu Y, Zhang Y, Wang Y (2018). ER-associated ubiquitin ligase HRD1 programs liver metabolism by targeting multiple metabolic enzymes. Nat Commun.

[29] Ippolito L, Sonveaux P, Chiarugi P (2022). Unconventional roles of lactate along the tumor and immune landscape. Trends Endocrinol Metab.

[30] Payen VL, Brisson L, Dewhirst MW, Sonveaux P (2015). Common responses of tumors and wounds to hypoxia. Cancer J.

[31] Viswanath P, Najac C, Izquierdo-Garcia JL, Pankov A, Hong C, Eriksson P, et al. Mutant IDH1 expression is associated with down-regulation of monocarboxylate transporters. Oncotarget. 2016;7. 10.18632/oncotarget.9006 PMC5085201 27144334

[32] Kirk P, Wilson MC, Heddle C, Brown MH, Barclay AN, Halestrap AP (2000). CD147 is tightly associated with lactate transporters MCT1 and MCT4 and facilitates their cell surface expression. EMBO J.

[33] Bhattacharya A, Qi L ER-associated degradation in health and disease—from substrate to organism. J Cell Sci. 2019;132. 10.1242/jcs.232850 PMC6918741 31792042

[34] Wei J, Harada BT, Lu D, Ma R, Gao B, Xu Y (2021). HRD1-mediated METTL14 degradation regulates m(6)A mRNA modification to suppress ER proteotoxic liver disease. Mol Cell.

[35] Parks SK, Mueller-Klieser W, Pouysségur J (2020). Lactate and acidity in the cancer microenvironment. Annu Rev Cancer Biol.

[36] Guan X, Rodriguez-Cruz V, Morris ME (2019). Cellular uptake of MCT1 inhibitors AR-C155858 and AZD3965 and their effects on MCT-mediated transport of L-lactate in murine 4T1 breast tumor cancer cells. AAPS J.

[37] Noble RA, Bell N, Blair H, Sikka A, Thomas H, Phillips N (2017). Inhibition of monocarboxyate transporter 1 by AZD3965 as a novel therapeutic approach for diffuse large B-cell lymphoma and Burkitt lymphoma. Haematologica..

[38] Puri S, Juvale K (2020). Monocarboxylate transporter 1 and 4 inhibitors as potential therapeutics for treating solid tumours: A review with structure-activity relationship insights. Eur J Med Chem.

[39] Fan Y, Wang J, Jin W, Sun Y, Xu Y, Wang Y (2021). CircNR3C2 promotes HRD1-mediated tumor-suppressive effect via sponging miR-513a-3p in triple-negative breast cancer. Mol Cancer.

[40] Jeon Y-J, Kim T, Park D, Nuovo GJ, Rhee S, Joshi P (2018). miRNA-mediated TUSC3 deficiency enhances UPR and ERAD to promote metastatic potential of NSCLC. Nat Commun.

[41] Wang W-F, Yan L, Liu Z, Liu L-X, Lin J, Liu Z-Y (2017). HSP70-Hrd1 axis precludes the oncorepressor potential of N-terminal misfolded Blimp-1s in lymphoma cells. Nat Commun.

[42] Guo X, Wang A, Wang W, Wang Y, Chen H, Liu X (2021). HRD1 inhibits fatty acid oxidation and tumorigenesis by ubiquitinating CPT2 in triple-negative breast cancer. Mol Oncol.

