# Supplementary material for: SYVN1-mediated ubiquitylation directs localization of MCT4 in the plasma membrane to promote the progression of lung adenocarcinoma
Source: Cell Death Dis. 2023 Oct 10;14(10):666. doi: 10.1038/s41419-023-06208-x (PMC10564934; doi:10.1038/s41419-023-06208-x)
Supplement: Supplementary file 1 — Supplementary Materials [file 41419_2023_6208_MOESM1_ESM.docx]

**Supplementary Materials and Figures**

SYVN1-mediated ubiquitylation directs localization of MCT4 in the plasma membrane to promote the progression of lung adenocarcinoma

Meng Zhao^1,2,3,4,#^, Chen Huang^1,2,3,4,#^, Lexin Yang^1,2,3,4,#^, Boyu Pan^2,3,4,5,#^, Shuting Yang^1,2,3,4^, Jiao Chang^1,2,3,4^, Yu Jin^1,2,3,4^, Gang Zhao^2,3,4,6^, Dongsheng Yue^2,3,4,7^, Shuo Qie^2,3,4,6^ and Li Ren^1,2,3,4,*^

^1^ Department of Clinical Laboratory, Tianjin Medical University Cancer Institute & Hospital, Tianjin, China.

^2^ National Clinical Research Center for Cancer, Tianjin, China.

^3^ Key Laboratory of Cancer Prevention and Therapy (Tianjin), Tianjin, China.

^4^ Tianjin's Clinical Research Center for Cancer, Tianjin, China.

^5^ Department of Molecular Pharmacology, Tianjin Medical University Cancer Institute & Hospital, Tianjin, China.

^6^ Department of Pathology, Tianjin Medical University Cancer Institute & Hospital, Tianjin, China.

^7^ Department of Lung Cancer, Tianjin Medical University Cancer Institute & Hospital, Tianjin, China.

**Supplementary Materials and Methods**

**Antibodies and reagents**

The following antibodies were used for immunoblot (IB) and immunofluorescence (IF) analyses: mouse anti-Flag (IB, 1:1000; IF, 1:200; F1804, Sigma‒Aldrich), rabbit anti-HA (IB, 1:1000; IF, 1:200; 3724S, CST), rabbit anti-His (IB, 1:1000; IF, 1:200; 12698S, CST), mouse anti-ubiquitin (IB, 1:1000; 3936S, CST), rabbit anti-ubiquitin (IF, 1:50; R26024, Zenbio), mouse anti-MCT4 (IB, 1:500; IF, 1:50; sc-376140, Santa Cruz Biotechnology), rabbit anti-SYVN1 (IB, 1:1000; IF, 1:50; 121294, Zenbio), mouse anti-β-actin (IB, 1:1000; A1978, Sigma‒Aldrich), and rabbit anti-Na+/k+ ATPase 1 (IB, 1:1000; 380790, Zenbio). Peroxidase-conjugated goat anti-mouse IgG (IB, 1:10000; ab205719) or goat anti-rabbit IgG (IB, 1:10000; ab6721), Alexa Fluor 488-conjugated anti-mouse IgG (IF, 1:200; ab150113), and Texas red-conjugated anti-rabbit IgG (IF, 1:1000; ab6719) were purchased from Abcam. Anti-Flag M2 affinity gel (A2220) and 3×Flag peptide (F4799) were purchased from Sigma‒Aldrich, NI-NTA Beads 6FF (SA004100) were obtained from Smart Lifescience, and cycloheximide (HY-12320) and MG132 (CS-0471) were purchased from MedChemExpress.

**Plasmids and transfection**

Flag-tagged MCT4 and HA-tagged SYVN1 were purchased from GeneChem. MCT4-ΔC (aa1-399) and MCT4-C only mutant (aa400-465) were constructed into the GV141 C-terminal Flag-tagged vector. N-terminal His-tagged ubiquitin was cloned into the pCDNA3.1 vector. shSYVN1s (target sequences, shSYVN1-1#: 5’-GCTCACGC CTACTACCTCAAA-3’, shSYVN1-2#: 5’-GACCGTGTGGACTTTATGGAA-3’) were cloned into the pLKO.1-puro vector. His-tagged CD147 was purchased from GeneChem. All the constructs were confirmed via DNA sequencing. Transfections were performed using polyethylenimine (PEI) reagent (Sigma) or Lipofectamine 3000 (Invitrogen).

**Cell culture and viral infection**

All cell lines were obtained from the National Collection of Authenticated Cell Cultures unless otherwise specified. HEK293T, HepG2, Hep3B, SK, HLE, HCT116, and DU145 cells were cultured in DMEM supplemented with 10% foetal bovine serum at 37 °C in 5% CO_2_. A549, H1752, H460, H1299, OVCAR3 and 786-O cells were cultured in RPMI 1640 supplemented with 10% foetal bovine serum at 37 °C in 5% CO_2_. Cell lines in which the SYVN1 gene was stably silenced (A549/H1752-shSYVN1) were generated from A549 and H1752 cells. To prepare retrovirus for the knockdown experiments, HEK293T cells were transfected with the pLKO.1-SYVN1-shRNA vector and the packaging vectors PSPAX2 and pMD2G using PEI reagent. Medium containing the virus was collected 48 h after transfection. A549 and H1752 cells were incubated with collected virus supernatants for 12 h with 8 µg/mL polybrene (Solarbio). Infected cells were selected with puromycin (Sigma‒Aldrich).

**Glucose consumption, lactate production and LDH activity**

A549/H1752-scramble and A549/H1752-shSYVN1 cells were cultured in RPMI-1640 (no phenol red) supplemented with 10% foetal bovine serum at 37 °C in 5% CO_2_. Cell supernatants were analysed at various time intervals with the glucose assay reagent (1707801, VITROS) and the lactic acid assay kit reagent (8433880, VITROS) by a VITROS 5600 automatic biochemical analyser (Ortho Clinical Diagnostics). Lactate and glucose quantification in the intracellular fraction were analysed with a glucose assay reagent (A154-1-1, Jianchengbio) and the lactic acid assay kit reagent (A019-2-1, Jianchengbio). The LDH activity kit (A020-1, Jianchengbio) was used according to the manufacturer’s instructions.

**Cellular oxygen consumption rate (OCR) and extracellular acidification rate (ECAR) measurement**

OCR and ECAR were measured using an XF24 Extracellular Flux Analyser (Seahorse Bioscience) as previously described [1]. In brief, pretreated SCLC cells were seeded in 24-well plates at a density of 1,000 cells/well and cultured overnight. Then, the cells were washed with either OCR medium (containing 4.5 g/L glucose, 2 mM glutamine and 1 mM pyruvate) or ECAR medium (containing 2 mM glutamine and no pyruvate or glucose) and incubated in a CO2-free incubator at 37 °C for 1 h to equilibrate the temperature and pH equilibration prior to loading into the XF24 apparatus. XF assays consisted of the following cycles: Mix (3 min), Wait (2 min), and Measure (3 min). The procedure included 3 basal rate measurements prior to the first injection and 3 rate measurements after each injection. ECAR was measured under baseline conditions and after treatment with glucose (100 mM), oligomycin (100 μM) and 2-deoxy glucose (2-DG; 500 mM). OCR was measured under baseline conditions and after treatment with oligomycin (100 μM), FCCP (100 μM) and rotenone/antimycin (50 μM). Values were normalized to 1 × 10^4^ cell counts. Values are presented as the mean ± standard error.

**Immunopuriﬁcation and silver staining**

The cells transfected with vector/Flag-MCT4 were immunopuriﬁed by anti-Flag M2 beads. Isolated proteins were separated in 8% SDS‒PAGE gel by electrophoresis. Silver staining was performed with the Pierce™ Silver Stain Kit (Thermo Scientific) following the manufacturer’s recommendations.

**Western blot**

Cells were treated with EBC lysis buffer containing 4% SDS, phenylmethylsulfonyl fluoride, and protease inhibitor cocktail and heated at 95 °C for 12 min. A BCA Protein Assay Kit (Solarbio) was used to quantify the total amounts of protein. Lysates were subjected to SDS/PAGE gel electrophoresis followed by transfer to PVDF membranes (Millipore). After blocking with 5% nonfat milk for 1 h, the membrane was incubated at 4 °C overnight with the relevant primary antibodies. The images were obtained with a Gel Imager (Tanon) and analysed with the Gel Image System (Tanon).

**Immunohistochemistry analysis**

In this study, 20 sets of NSCLC tissue samples were obtained from patients at Tianjin Medical University Cancer Institute & Hospital. The studies involving human specimens were reviewed and approved by the Tianjin Medical University Cancer Institute & Hospital (No.: bc2019038). All participants signed an informed consent form. Immunohistochemistry was performed on serial tissue microarrays of NSCLC purchased from Shanghai Outdo Biotech and on tumour tissues from the mouse model. Immunohistochemistry and histological analysis of animal tissues were carried out at Bioss Biotechnology Co., Ltd. The intensity and density of positive cells were two important evaluation parameters used for scoring. The intensity of positive cells was evaluated based on the colour of the positive cells, which was classified as 0 (no staining), 1 (weak), 2 (moderate), and 3 (strong). The density of positive cells was sorted into four levels: 0 (staining ≤ 5%), 1 (5% < staining ≤25%), 2 (25% < staining ≤50%), 3 (50% < staining ≤75%) and 4 (staining＞75%). According to the total scores, which were generated by adding the scores for the intensity and density of positive cells, the levels of staining were graded as ‘–’ (score 0), ‘+’ (score 1–4), ‘++’ (score 5–8), and ‘++’ (score 9-12). Cases of ‘–’ and ‘+’ were assigned to the group of low expression levels, whereas cases of ‘++’ and ‘+++’ were assigned to the group of high expression levels.

**Immunoﬂuorescence staining**

The cells were cultured on coverslips in 24-well plates, washed three times with PBS, fixed for 15 min at room temperature with 4% paraformaldehyde and permeabilized with 0.2% Triton X-100 for 10 min. Following permeabilization, the cells were blocked by incubation for 30 min with 0.5% goat serum in PBS, and then the cells were incubated overnight with specific primary antibodies. After the cells were washed with PBS three times, they were incubated in 0.5% goat serum with secondary antibodies for 1 h at room temperature. After washing, DAPI was used to stain the cell nuclei. The slides were mounted with mounting solution and observed under a laser scanning confocal microscope (Zeiss LSM-880).

**qRT‒PCR**

Total mRNA was isolated using a Total RNA Extraction Kit (Solarbio), and 2 µg of RNA was used to synthesize cDNA using the ReverAid First Strand cDNA synthesis Kit (Thermo Fisher Scientific) according to the manufacturer’s protocol. Real-time PCR was performed using 2×SYBR Green qPCR Master Mix (Bimake) in an ABI 7500 Real-Time PCR system (Applied Biosystems). All gene expression levels were normalized against the corresponding levels of GAPDH. Sequences of primers used in this study: MCT4 forwards, 5’-CGGCTTTGTGCTTTACGCC-3’, MCT4 reverse, 5’-GCTGAAGAGGTAGACGGAGTA-3’; SYVN1 forwards, 5’-AGCCTGCGTAACATCCACAC-3’, SYVN1 reverse, 5’-AGTTGACTGAAGTGGCAGGC-3’; GAPDH forwards, 5’-GTCTCCTCTGACTTCAACAGCG-3’, GAPDH reverse, 5’-ACCACCCTGTTGCTGTAGCCAA-3’.

**Colony formation assay**

A549/H1752 scramble and shSYVN1 cells were seeded into 6‐well plates at a density of 500 cells per well using RPMI‐1640 supplemented with 10% FBS. The medium was replaced every two days. When most cell clumps achieved > 50 cells, as observed under a microscope (Olympus), the colonies were then fixed with 4% paraformaldehyde for 1 h, stained with crystal violet (Solarbio), and counted.

**Cell viability assay**

Cell viability was analysed via a Cell Counting Kit (ZETA). Briefly, A549/H1752 scramble and shSYVN1 cells were plated in a 96-well plate at a density of 1000 cells per well and cultured overnight. Ten microlitres of CCK-8 solution was added to each well of the plate, the cells were incubated at 37 °C for 2 h, and the optical density at 450 nm was measured. Similar assays were performed after 24, 48 and 72 hours.

**EdU Cell Proliferation assay**

EdU staining was carried out with an EdU Cell Proliferation Image Kit (KTA2030, Abbkine) according to the manufacturer’s instructions. Briefly, A549/H1752 scramble and shSYVN1 cells were fixed and then permeabilized before staining. A Click-iT reaction mixture containing fluorescently labelled EdU was added, and the cells were incubated for 30 min. The stained samples were observed and analysed under a fluorescence microscope (Olympus), and the density of positive cells was analysed using ImageJ.

**Isolation of Plasma Membrane Protein**

Plasma membrane and cell fractions were isolated using the Minute^TM^ Plasma Membrane Protein Isolation and Cell Fractionation Kit (SM-005, Invitrogen). All procedures were performed on ice and followed the manufacturer's recommended protocols. Then, the plasma membrane isolation was dissolved in Minute^TM^ Denaturing Protein Solubilization Reagent (WA-009, Invitrogen). Proteins were detected by immunoblotting using an anti-MCT4 antibody.


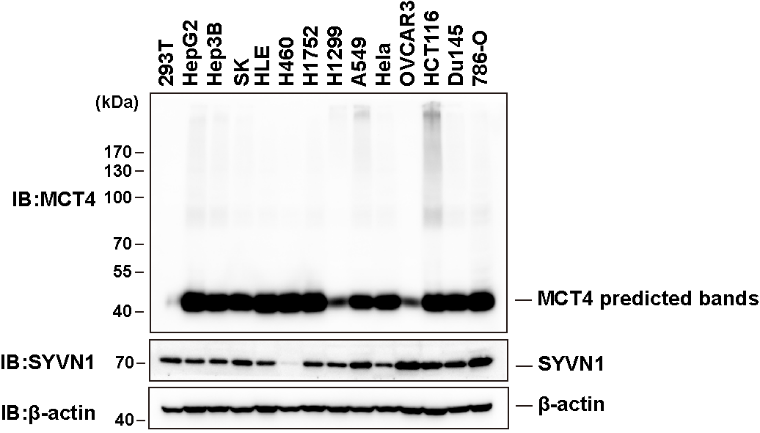


**Supplementary Fig. 1** Western blot detects a smeared pattern above the predicted MCT4 bands and SYVN1 expression in listed cells. The membrane is the same as in Fig. 1A.


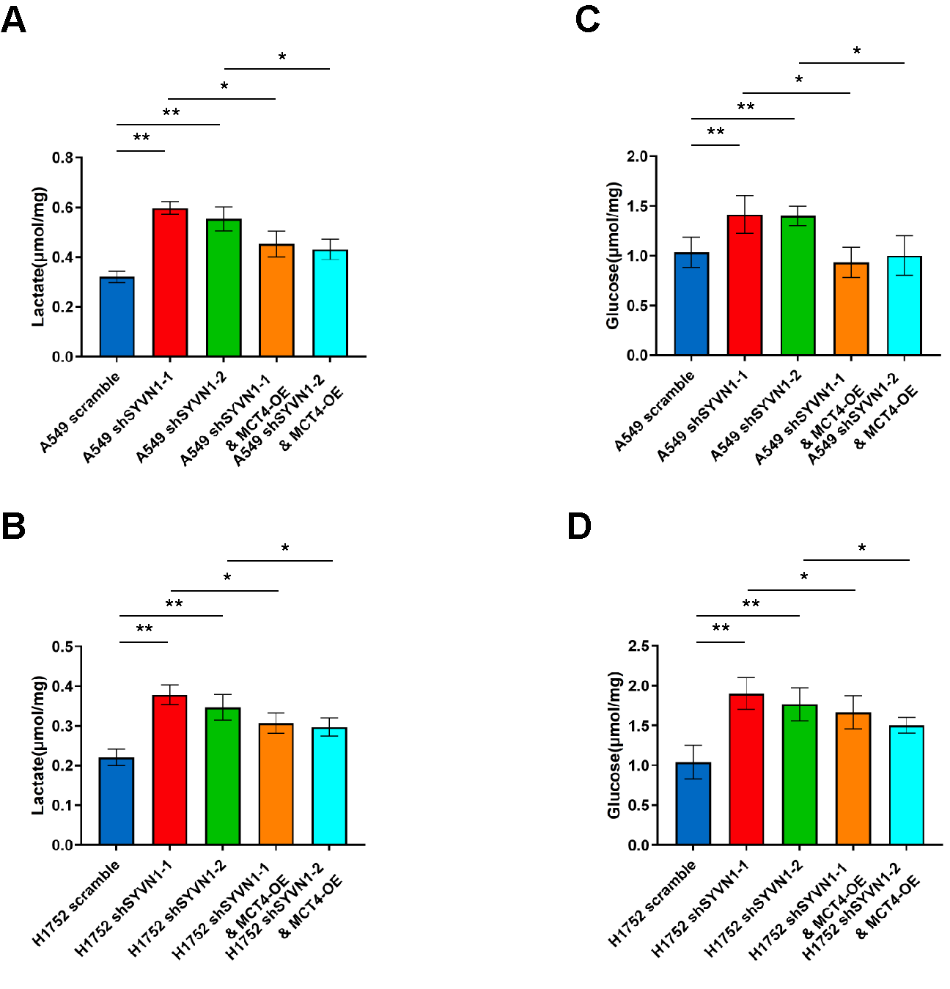


**Supplementary Fig. 2** **A-D** Ectopic expression of MCT4 partially reverses intracellular lactate (A and B) and glucose (C and D) levels in A549 and H1752 cells infected with scramble or shRNAs targeting SYVN1. Data are presented as the mean ± SD (**P*<0.05, ***P*<0.01, n=5, two-tailed unpaired t tests).


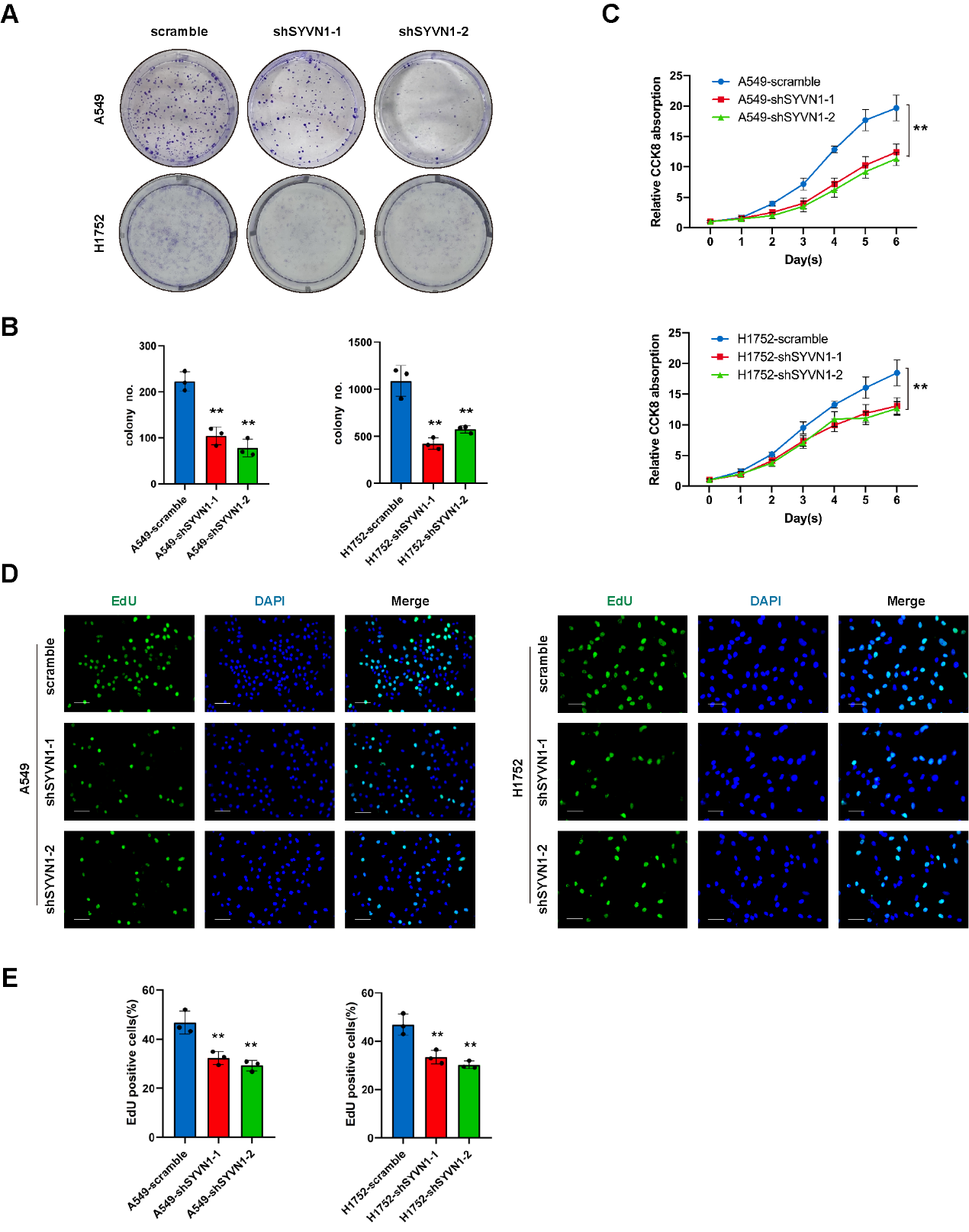


**Supplementary Fig. 3** SYVN1 promotes LUAD proliferation. **A, B** SYVN1 knockdown reduces colony formation. A549 and H1752 cells were infected with lentiviruses carrying scramble control shRNA (scramble) or shRNAs targeting SYVN1. (B) Data are quantified and presented as the mean ± SD (**P*<0.05, ***P*<0.01, n = 3, two-tailed unpaired t test). **C** SYVN1 knockdown compromises cell proliferation. CCK8 assays for the growth of A549 and H1752 cells infected with lentiviruses carrying scramble control shRNA (scramble) or shRNAs targeting SYVN1. Data are presented as the mean ± SD (***P*<0.01, n = 3, two-way ANOVA). **D, E** SYVN1 knockdown suppresses cell proliferation. EdU (5-ethynyl-2’- deoxyuridine) incorporation assays of A549 and H1752 cells infected with lentiviruses carrying scramble control shRNA (scramble) or shRNAs targeting SYVN1. (E) Data are quantified and presented as the mean ± SD (**P*< 0.05, ***P*<0.01, n = 3, two-tailed unpaired t test). Scale bar: 50 μm.


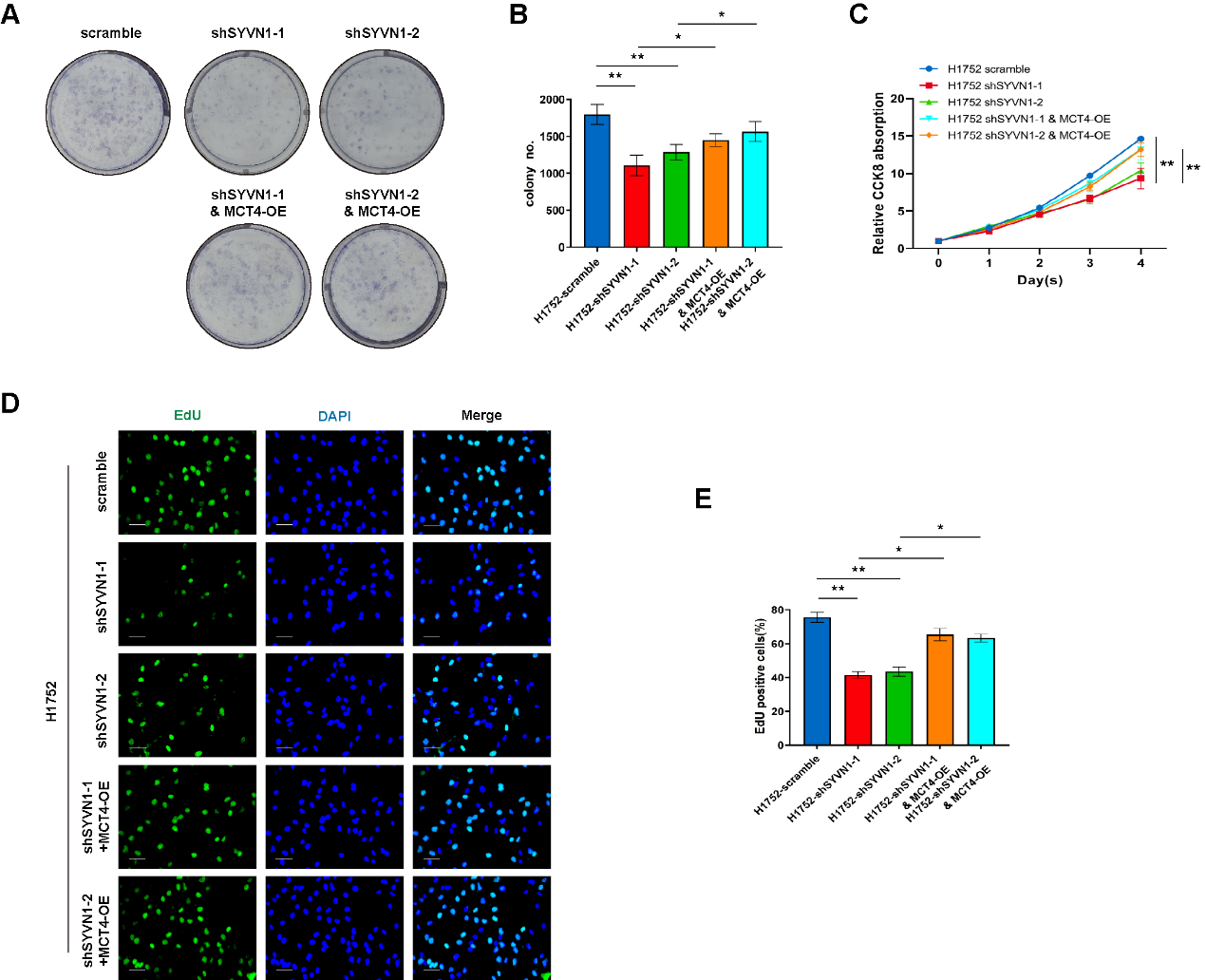


**Supplementary Fig. 4** Ectopic expression of MCT4 reverses the SYVN1 knockdown-induced inhibition of cell proliferation. **A, B** Colony formation of SYVN1 scramble and SYVN1 knockdown with ectopic expression of MCT4 in H1752 cells. (B) Data are quantified and presented as the mean ± SD (**P*<0.05, ***P*<0.01, n = 5, two-tailed unpaired t test). **C** CCK8 assays of SYVN1 scramble and SYVN1 knockdown with ectopic expression of MCT4 in H1752 cells. Data are presented as the mean ± SD (**P*<0.05, ***P*<0.01, n = 5, two-way ANOVA). **D, E** EdU incorporation assays of SYVN1 scramble and SYVN1 knockdown with ectopic expression of MCT4 in H1752 cells. (E) Data are quantified and presented as the mean ± SD (**P*< 0.05, ***P*<0.01, n = 6, two-tailed unpaired t test). Scale bar: 50 μm.


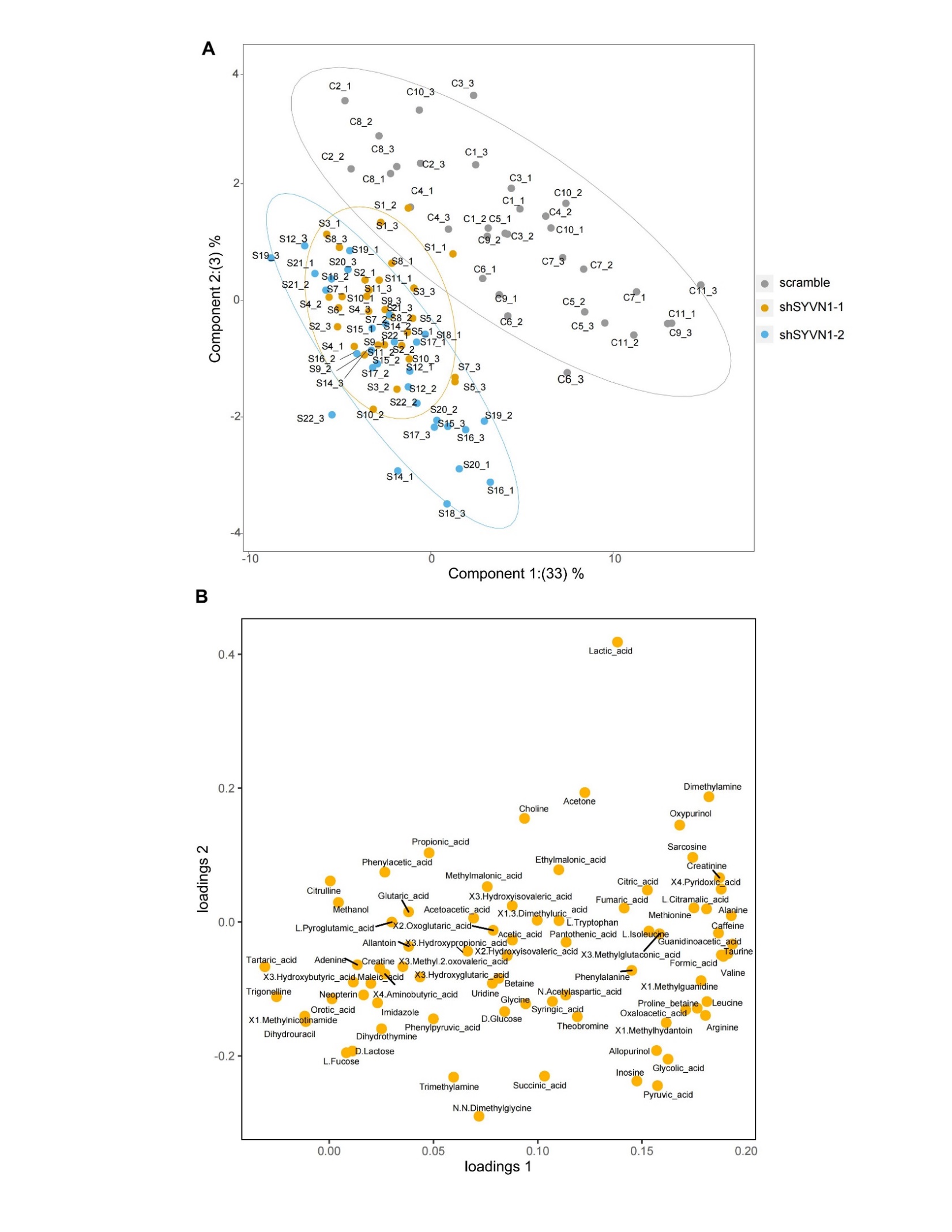


**Supplementary Fig. 5 A, B** Metabolites present in tumour tissues isolated from A549 cells infected with lentiviruses carrying scramble control shRNA (scramble) or shRNAs targeting SYVN1 were detected by NMR analysis. The PLS-DA score plot (A) shows the distinct trend followed by the two groups, and the PLS-DA load diagram (B) shows the contribution of the different metabolites to the discrimination between the two groups.

**SUPPLEMENTARY REFERENCE**

1. Zhao M, Liu Y, Chang J, Qi J, Liu R, Hou Y, et al. ILF2 cooperates with E2F1 to maintain mitochondrial homeostasis and promote small cell lung cancer progression. Cancer Biol Med. 2019;16(4):771-83.
