# Supplementary material for: SYVN1-mediated ubiquitylation directs localization of MCT4 in the plasma membrane to promote the progression of lung adenocarcinoma
Source: Cell Death Dis. 2023 Oct 10;14(10):666. doi: 10.1038/s41419-023-06208-x (PMC10564934; doi:10.1038/s41419-023-06208-x)
Supplement: Supplementary file 5 — Original Data File [file 41419_2023_6208_MOESM5_ESM.pdf]

Full length uncropped original western blots

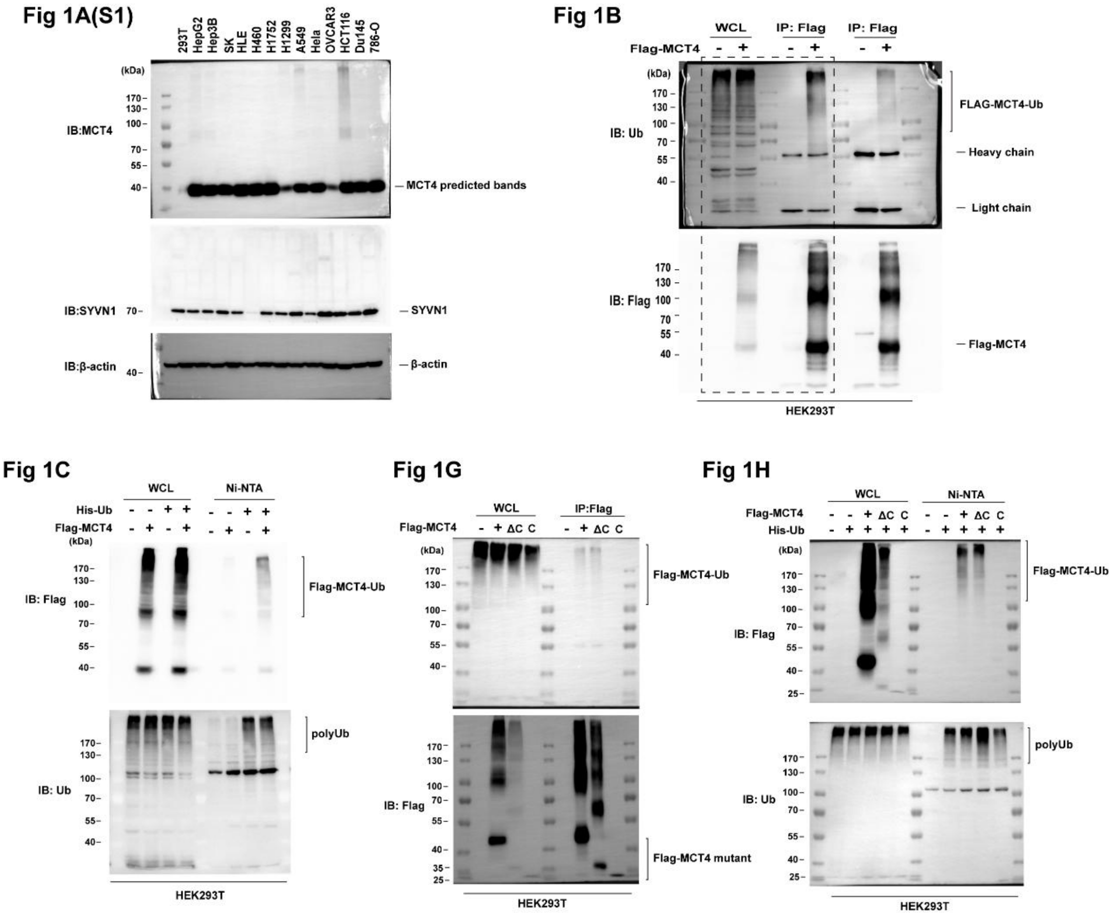

Original figure 1

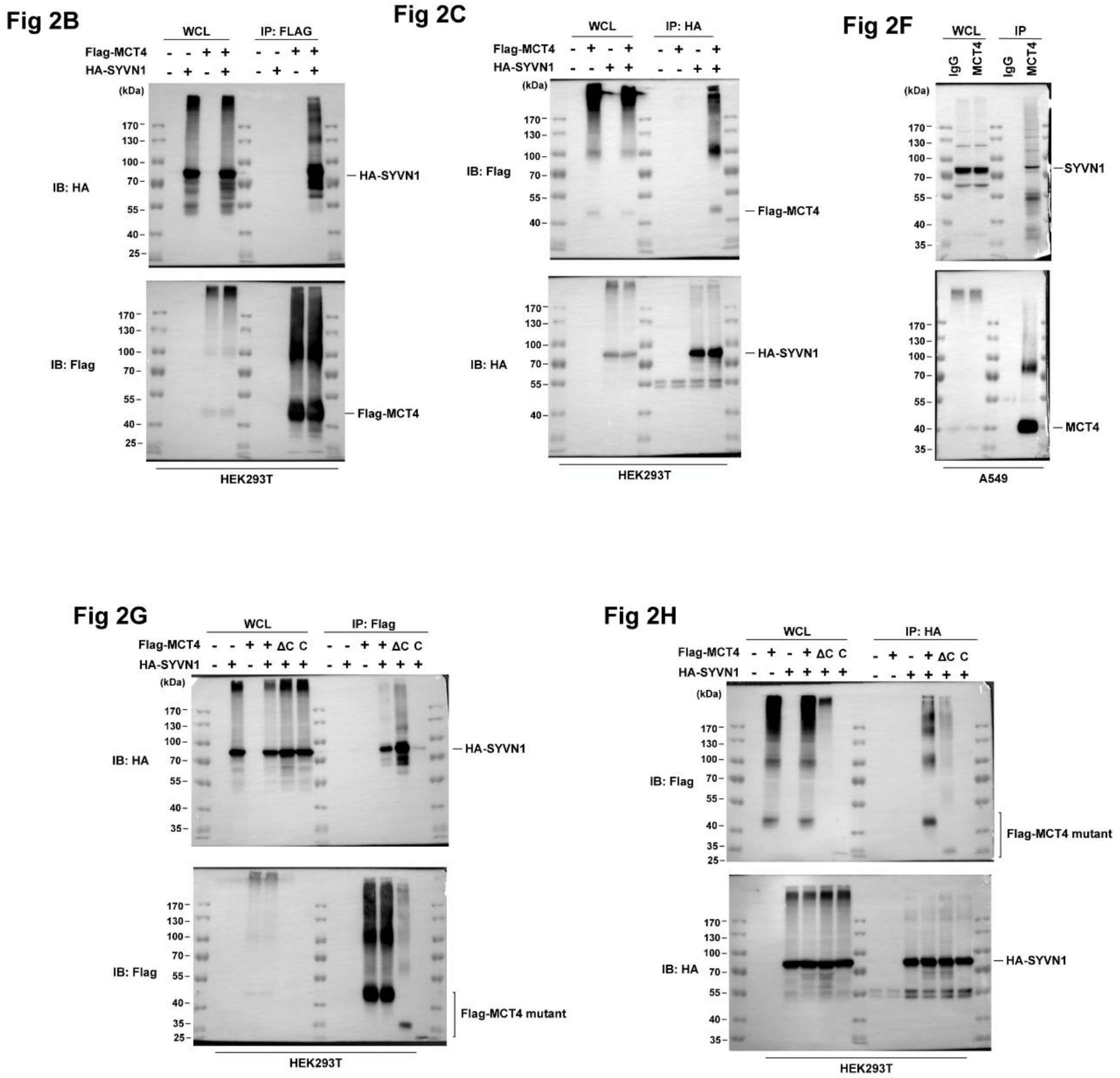

Original figure 2

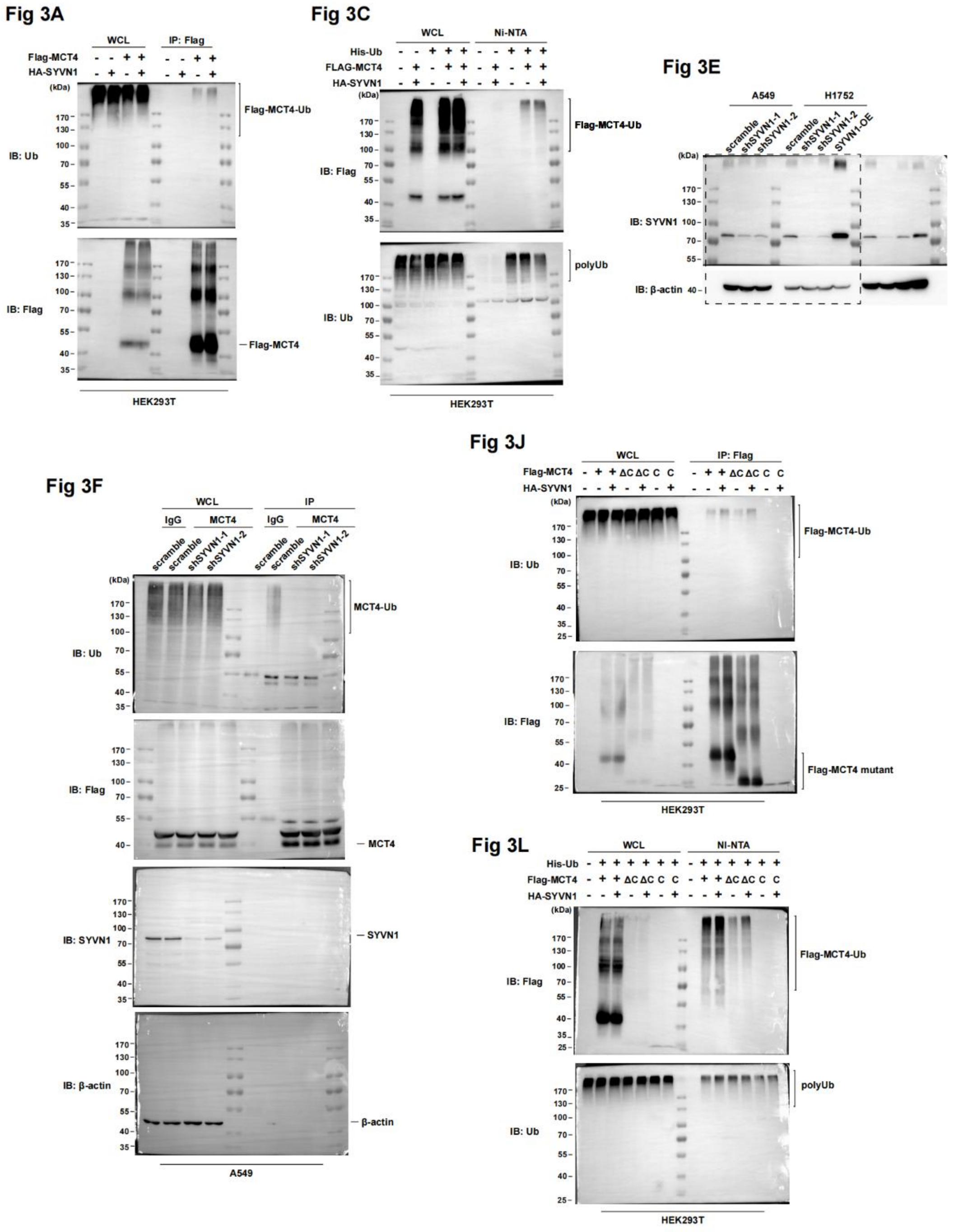

Original figure 3

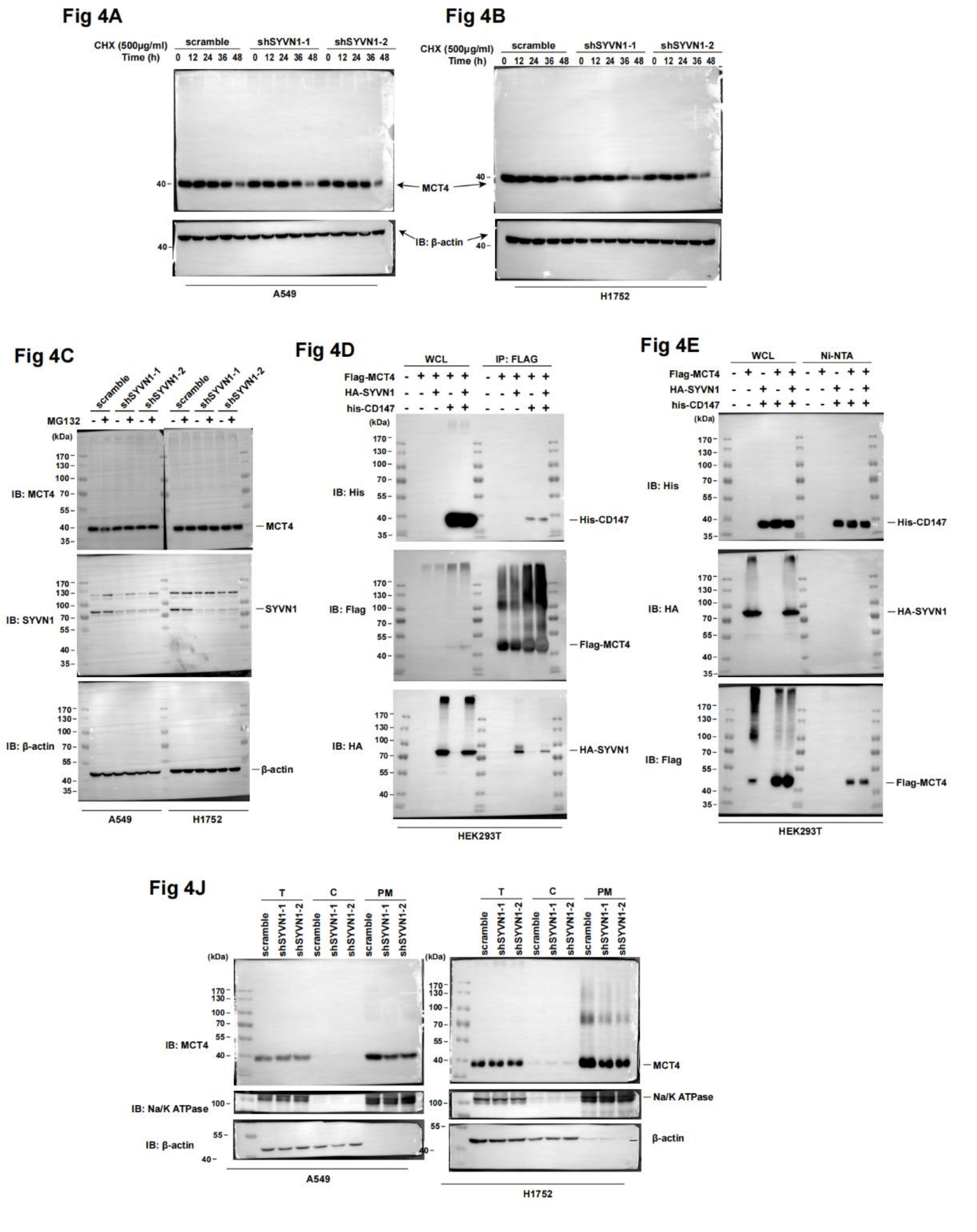

Original figure 4
